# Adaptation to spindle assembly checkpoint inhibition through the selection of specific aneuploidies

**DOI:** 10.1101/gad.350182.122

**Published:** 2023-03-01

**Authors:** Manuel Alonso Y Adell, Tamara C. Klockner, Rudolf Höfler, Lea Wallner, Julia Schmid, Ana Markovic, Anastasiia Martyniak, Christopher S. Campbell

**Affiliations:** Department of Chromosome Biology, Max Perutz Laboratories, University of Vienna, Vienna Biocenter (VBC), A-1030 Vienna, Austria

**Keywords:** aneuploidy patterns, chromosomal instability, spindle assembly checkpoint, drug resistance mechanisms, MPS1

## Abstract

Both the presence of an abnormal complement of chromosomes (aneuploidy) and an increased frequency of chromosome missegregation (chromosomal instability) are hallmarks of cancer. Analyses of cancer genome data have identified certain aneuploidy patterns in tumors; however, the bases behind their selection are largely unexplored. By establishing time-resolved long-term adaptation protocols, we found that human cells adapt to persistent spindle assembly checkpoint (SAC) inhibition by acquiring specific chromosome arm gains and losses. Independently adapted populations converge on complex karyotypes, which over time are refined to contain ever smaller chromosomal changes. Of note, the frequencies of chromosome arm gains in adapted cells correlate with those detected in cancers, suggesting that our cellular adaptation approach recapitulates selective traits that dictate the selection of aneuploidies frequently observed across many cancer types. We further engineered specific aneuploidies to determine the genetic basis behind the observed karyotype patterns. These experiments demonstrated that the adapted and engineered aneuploid cell lines limit CIN by extending mitotic duration. Heterozygous deletions of key SAC and APC/C genes recapitulated the rescue phenotypes of the monosomic chromosomes. We conclude that aneuploidy-induced gene dosage imbalances of individual mitotic regulators are sufficient for altering mitotic timing to reduce CIN.

## Abstract (precis)

In this study, Adell et al. investigated the bases of the selection of certain aneuploidy patterns in tumors, and by establishing time-resolved long-term adaptation protocols, they found that human cells adapt to persistent spindle assembly checkpoint (SAC) inhibition by acquiring specific chromosome arm gains and losses. They conclude that aneuploidy-induced gene dosage imbalances of individual mitotic regulators are sufficient for altering mitotic timing to reduce CIN.

The faithful segregation of replicated genetic material to daughter cells is a fundamental requirement of all living beings. Erroneous chromosome segregation during mitosis results in gains and losses of chromosomes and chromosome arms, resulting in aneuploidy. An increased rate of such chromosome missegregation events is termed chromosomal instability (CIN). Aneuploidy is associated with growth abnormalities and inviability in many organisms (for reviews, see Torres et al. 2008; Williams and Amon 2009). In humans, all autosomal chromosome losses are embryonic lethal, and only a few chromosome gains, such as trisomy 21, are compatible with life (Hassold and Hunt 2001). On the cellular level, aneuploidy results in dosage changes of hundreds of genes at once, leading to a wide variety of phenotypes including proteotoxic stress, genome instability, and cell cycle arrest (for reviews, see Zhu et al. 2018; Chunduri and Storchová 2019).

Despite these adverse effects, aneuploidy is found in ∼90% of solid tumors and ∼70% of hematopoietic cancers, making it one of the most common types of genetic alterations in cancer (Weaver and Cleveland 2006). Aneuploidy and CIN are associated with tumor progression and metastasis formation (Carter et al. 2006; McGranahan et al. 2012; Shukla et al. 2020). Experiments from yeast to human cells have shown that certain aneuploidies can be advantageous under specific stress conditions (Selmecki et al. 2006; Rutledge et al. 2016; Ravichandran et al. 2018; Salgueiro et al. 2020). Recent studies demonstrated that temporary induction of CIN confers resistance to chemotherapeutic drugs (Ippolito et al. 2021; Lukow et al. 2021). However, the selective forces underlying the retention of specific aneuploidies are currently not well understood.

Cancers have characteristic aneuploidy patterns. For example, certain aneuploidies, like the gain of chromosome arms 8q and 20q, are highly frequent across many different cancer types (Beroukhim et al. 2010). Other aneuploidies are only prevalent in specific cancer types, such as chromosome arm 3p loss in squamous cancers or 13q gain in gastrointestinal tumors (Taylor et al. 2018). The complexity of cancer karyotypes makes it difficult to determine the selective advantage of specific aneuploidies. However, many studies have shown interesting correlations in aneuploidy patterns. For example, a seminal study indicated that the distribution of tumor suppressors and oncogenes across chromosomes can predict whether a particular chromosome is more likely to be gained or lost (Davoli et al. 2013). Recent breakthroughs in experimental models for Ewing sarcoma in mice and human cells have found a partial contribution in the *Rad21* and *Myc* genes for the selection of chromosome 8 trisomy (Su et al. 2021). However, there are currently very few bottom-up methods to identify the adaptive advantage of specific aneuploidies in human cells and identify the underlying responsible genes.

The sources of CIN in cancer have long been elusive. Although many potential sources of CIN have been identified, such as supernumerary centrosomes, whole-genome duplication, and replication stress, the degree to which each of these abnormalities contributes to chromosome missegregation is still currently unclear (for review, see Sansregret et al. 2018). The most commonly observed type of chromosome missegregation in cancer cells is lagging chromosomes from merotelic attachments, wherein a single sister chromatid is attached to microtubules emanating from both spindle poles (Thompson and Compton 2008). More recently, unaligned chromosomes that do not converge at the metaphase plate have also been shown to contribute to missegregation in cancer cells (Gomes et al. 2022). Ordinarily, such missattachments would be destabilized, and spindle assembly checkpoint (SAC) would delay anaphase until all chromosomes have been fully aligned. The SAC is a surveillance mechanism monitoring accurate and timely attachment of chromosomes to the mitotic spindle (for reviews, see Musacchio and Salmon 2007; Silva et al. 2011; Pachis and Kops 2018; Lara-Gonzalez et al. 2021). SAC activation inhibits the anaphase-promoting complex/cyclosome (APC/C), preventing cells from entering anaphase until all of the chromosomes are attached to spindle microtubules (Watson et al. 2019). Despite the high rates of CIN, most cancer cells have a functional SAC (Tighe et al. 2001). However, disruption of SAC activity can lead to tumorigenesis in mice, demonstrating the importance of the SAC as a tumor suppression mechanism (Sotillo et al. 2007). Interestingly, a number of oncogenic viruses down-regulate the activity of SAC components (Jin et al. 1998; Sun et al. 2014; Shirnekhi et al. 2017). However, it is currently unclear whether and how persistent SAC down-regulation influences complex aneuploid karyotype formation in human cells.

In this work, we used a time-resolved adaptation assay based on long-term inhibition of the SAC kinase MPS1 to analyze how complex aneuploid karyotypes change over time in response to CIN. We found that complex karyotypes converge on very similar aneuploidy patterns. These complex karyotypes are then refined toward smaller copy number alterations (CNAs). Intriguingly, the frequencies of chromosome gains in the adapted cells correlate with those seen in cancer, suggesting general advantages that are largely independent of the cell type and cellular environment. We then used CRISPR/Cas9-based engineering of monosomic chromosomes to determine the genetic bases behind frequently acquired aneuploidies. We identified specific monosomies that directly rescue SAC inhibition. We show that these monosomies increase the mitotic duration and that changing the dosage of single genes is sufficient to reduce CIN. Together, these results demonstrate that alterations in single genes on aneuploid chromosomes are sufficient to affect mitotic timing and suppress SAC inhibition.

## Results

### Long-term MPS1 inhibition leads to selection of specific aneuploidies and karyotype refinement over time

**Figure 1.**
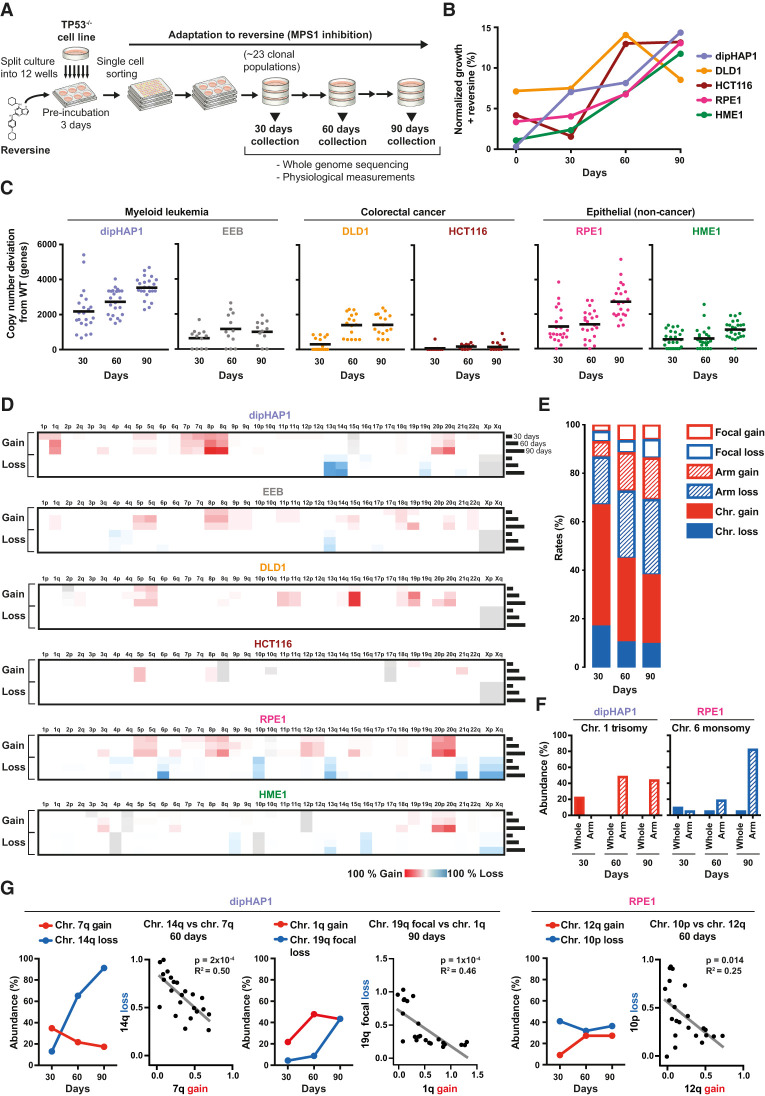
Long-term MPS1 inhibition leads to the selection of specific aneuploidy types and karyotype refinement over time. (*A*) Schematic overview of the reversine adaptation process. (*B*) Average of the growth in reversine of all populations of each cell line at 30, 60, and 90 d as measured using colony formation assays. Time point 0 represents growth of unadapted parental cell lines. The percentages are normalized to the growth of the parental cell lines in the absence of reversine. (*C*) Total copy number changes (of whole chromosomes, arms, and segments) of reversine-adapted populations at 30, 60, and 90 d derived from Illumina sequencing read frequencies. Copy number alterations that were already present in the parental cell lines before adaptation are excluded. The black line represents the mean number of genes with altered copy numbers per adapted cell line. (*D*) Heat maps depicting the mean percentage of copy number changes in chromosome arms from all populations of each adapted cell line at 30, 60, and 90 d. Cell line-inherent aneuploidies that were present at time point 0 before the adaptation are colored in gray. For all figures, chromosome gain is shown in red, and chromosome loss is shown in blue. (*E*) Quantification of the relative percentage of copy number changes of whole chromosomes (filled), arms (dashed lines), and segments (focal changes; empty) across all reversine-cultivated cell lines at 30, 60, and 90 d. (*F*) The percentage of adapted cell lines with whole and chromosome arm copy number changes of chromosome 1 in dipHAP1 and chromosome 6 in RPE1, respectively. (*G*) Comparisons of the abundance of dipHAP1- or RPE1-adapted populations with specific chromosome losses or gains over time and the corresponding correlations between the chromosome copy number (0 indicates no loss/gain, and 1 indicates chromosome loss/gain; *X*-axis) for individual populations at 60 or 90 d, respectively. *P*-values are from *F*-tests.

To study how human cell karyotypes change over time in the presence of high rates of chromosome missegregation, we continuously treated *p53*-deleted cell lines with the MPS1 inhibitor reversine over 30, 60, and 90 d (Fig. 1A). Reversine was added at concentrations high enough to act simultaneously as a CIN-inducing agent and a strong selective pressure (Supplemental Fig. S1A,B). Before initiating the adaptation process, we first engineered human cell lines suitable for observing adaptation to high rates of CIN. We started with six near-diploid human cell lines that comprise three categories: myeloid leukemia (diploidized HAP1 -[referred to here as dipHAP1] and EEB), colorectal cancer (HCT116 and DLD1), and epithelial noncancer (hTERT-RPE1 and hTERT-HME1). Near-diploid cell lines were chosen to reduce the likelihood of pre-existing CIN-tolerating mutations and to simplify the karyotype analyses. In cell lines that did not already have a *p53* deletion, we homozygously deleted *p53* so that they could continue to propagate despite ongoing chromosome missegregation (Supplemental Fig. S1C; Santaguida et al. 2017). In addition, the vast majority of cancers with CIN have dysfunctional p53 pathways, making *p53*-deleted cells a good model for adaptation to CIN. The homozygous deletion of *p53* in the cell lines led to no or very few karyotypic changes compared with wild-type counterparts, with the exception of HME1, which displayed the most initial chromosome aberrations (Supplemental Fig. S1D,E; Supplemental Table S2). Most cell lines showed a cell type-dependent decrease in their capacity to arrest at the G1/S transition following *p53* deletion, as previously described (Supplemental Fig. S1F; Kastan et al. 1991; Hartwell and Kastan 1994). Notably, the capacity of wild-type DLD1 and HME1 cell lines to arrest at G1 was generally low and did not significantly change upon *p53* inactivation. In DLD1, we attribute this to the presence of the *p53*^*S241F*^ mutation, which fails to activate the p21 pathway (Sur et al. 2009). In HME1, this could be connected to the repression of the G1/S regulator p16^INK4a^, which has been described to occur in later passages of this cell type (Shapiro et al. 1998; Kim et al. 2002).

To obtain cycling cells adapted to long-term MPS1 inhibition, multiple cultures of the parental *p53*-deleted cell line were first preincubated in medium containing reversine for 3 d and then subjected to single-cell sorting to obtain clonal populations. At least 20 independent clonal populations were then cultivated in the presence of reversine for a period of 90 d. In parallel, six populations from each cell line were propagated in the absence of reversine as a control. At each 30-d time interval, cell populations were analyzed using a combination of low-coverage whole-genome sequencing and fitness measurements. This simultaneous adaptation of many independent cultures allowed us to identify recurring karyotypic changes resulting from positive selection.

Most of the adapted cell populations showed a gradual increase in reversine resistance over time as measured by colony formation assays. Proliferation of cells in reversine increased from 0.4%–7.2% relative to untreated cells before the adaptation (time point 0) to ∼13%–15% at 90 d (Fig. 1B). Proliferation of the EEB cell line was not measured, as the nonadherent nature of this cell line prevented colony formation. To determine the types of somatic copy number alterations acquired in the adapted cell lines, we measured copy number changes using low-coverage next-generation sequencing (NGS). Copy number was measured relative to the untreated parental population in 7.5-Mb intervals. From this analysis, we identified copy number changes of entire chromosomes and chromosome arms as well as focal copy number alterations. We define aneuploidy as the gain or loss of either whole chromosomes or whole chromosome arms (Ben-David and Amon 2020). The degree of aneuploidy varied considerably between cell populations of the same cell type and even more so across cell types (Fig. 1C,D; Supplemental Fig. S2). On average, the dipHAP1 and RPE1 populations exhibited the highest degree of aneuploidy with 18% and 14% of the genome being aneuploid at 90 d, respectively. In contrast, the HCT116 populations showed the least amount of aneuploidy at ∼1% of the genome on average. We conclude that aneuploidy formation in response to long-term MPS1 inhibition is highly cell line-dependent.

We next measured which types of copy number changes (whole chromosome, arm, or focal) were most prevalent in the adapted populations and how this changed over time. For all cell lines combined, whole-chromosome aneuploidies made up >60% of all CNAs at the 30-d time point (Fig. 1E). Over time, the relative amount of whole-chromosome aneuploidy decreased as arm and focal CNAs both increased. One reason for this trend can be seen in specific aneuploidies that changed from whole-chromosome CNAs at the 30-d time point into arm aneuploidies at later stages. This is exemplified by chromosome 1 trisomy turning into the gain of only the q-arm in dipHAP1 cells and chromosome 6 monosomy turning into monosomy of just the p-arm in RPE1 cells (Fig. 1F). The transition to smaller regions over time is also apparent from the increase in focal changes from 7% at 30 d to 14% at the 90-d time point. Notably, the increase in arm and focal aneuploidies was also seen when the acrocentric chromosomes were considered as whole chromosomes rather than chromosome arms (Supplemental Fig. S3A,B). The complete lack of whole-chromosome and arm-level losses in DLD1 and HCT116, respectively, indicated fundamental differences in how the diverse cell lines adapted to the drug (Supplemental Fig. S3B). We conclude that early on in adaption to CIN, whole-chromosome changes are primarily acquired. At later stages, adapted populations develop more refined karyotypes by gaining and losing smaller chromosomal areas.

### Correlations between aneuploidies over time

Although many aneuploidies increased in frequency over the course of the experiment, we were surprised to see that some aneuploidies that were frequently acquired early on in the adaptation process decreased in prevalence at later time points. These include the gain of chromosome 7q and chromosome 1q in dipHAP1 cells and the loss of chromosome 10p in RPE1 cells (Fig. 1D,G; Supplemental Fig. S2). We hypothesized that these decreases could be due to incompatibilities with other, more beneficial chromosomes. For each of the examples mentioned above, we identified strong negative correlations with other aneuploidies that show corresponding increases over time. Aneuploidy of chromosome 7q, 1q, and 10p was each mutually exclusive with chromosome 14q monosomy, chromosome 19q partial loss, and chromosome 12q gain, respectively (Fig. 1G). For chromosome 19q, we observed a combination of whole-arm monosomies and deletion of only the last 7.5 Mb of the chromosome (Supplemental Fig. S3C). The anticorrelation with chromosome 1q gain was compared with both forms of 19q loss combined. These patterns indicate that distinct aneuploidies are lost when more beneficial aneuploidies take over the population. Indeed, for chromosome 7q trisomy versus chromosome 14q monosomy and for chromosome 10p monosomy versus chromosome 12q trisomy, the aneuploidy that emerged later in adaptation correlated better with growth in reversine (Supplemental Fig. S3D). We conclude that optimized aneuploid karyotypes develop over time through both the gradual selection of highly beneficial aneuploidies and the loss of less beneficial aneuploidies acquired early on in the adaptation process.

### Cell lines adapted to reversine converge on recurrent aneuploidy patterns

**Figure 2.**
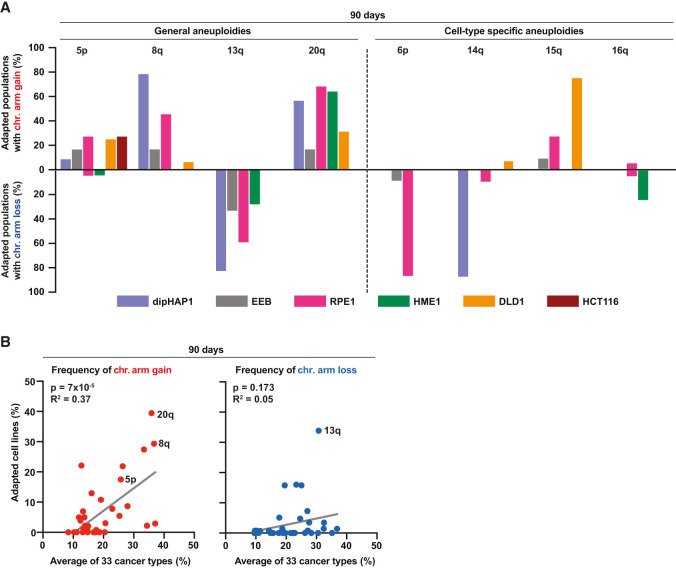
Reversine-cultivated cell populations converge on recurrent aneuploidy patterns that partially correlate with those seen in cancer. (*A*) Percentage of adapted populations containing specific chromosome arm gains (upward) and chromosome arm losses (downward) among all populations of the different cell lines at the 90-d time point. (*B*) Correlation between the frequencies of chromosome arm gains (*left*; red dots) and losses (*right*; blue dots) in reversine-adapted cell populations after 90 d of adaptation and 33 different cancer types. The frequency of chromosome arm gains or losses of the adapted cell lines represents the mean of the frequencies from all six different adapted cell lines. *P*-values are from *F*-tests.

We next determined which specific aneuploidies were most enriched at the end of the adaptation. For these analyses, we focused on whole-chromosome and arm-level CNAs, as these were most frequently observed. Cultivation of cell populations in the absence of reversine did not change karyotypes during the 90 d, with the exception of the loss of chromosome Xp in dipHAP1 (Supplemental Fig. S4A). By the 90-d time point, the vast majority of reversine-adapted populations had acquired recurring aneuploidies in all cell lines except for HCT116. Importantly, none of the cell populations underwent whole-genome duplication (WGD) during the 90 d of adaptation, demonstrating that WGD did not drive reversine resistance in these experiments (Supplemental Fig. S4B). There were some aneuploidies that were acquired in adapted populations across multiple cell lines. These include the gain of chromosomes 5, 8, and 20 in three or four out of the six adapted cell lines (Figs. 1D, 2A). In addition, chromosome 13q was frequently lost in four cell lines. These results suggest that there are certain aneuploidies that provide a general benefit to reversine-induced CIN. In addition to the general aneuploidies, we also observed cell type-specific karyotypic changes. Examples of aneuploidies that only frequently occurred in one cell line include chromosome 15q gain in DLD1, 6p loss in RPE1, 14q loss in dipHAP1, and 16q loss in HME1. Each of these alterations was present in >80% of the independently adapted cultures at the 90-d time point (Fig. 2A). Notably, during the adaptation period, some populations reverted cell line-specific aneuploidies that were present at time point 0 (Supplemental Fig. S4C). These include the loss of focal chromosome 3p trisomy in HME1 and chromosome X disomy in dipHAP1. The dipHAP1 cell line was generated through diploidization of male haploid chronic myeloid leukemia cells that lost chromosome Y. Therefore, dipHAP1 cells are aneuploid for chromosome X because they have two active copies. We conclude that both cell line-dependent and -independent aneuploidies are selected for during the adaptation to CIN.

### The patterns of chromosome gains in reversine-adapted populations correlate with frequencies observed in cancer

To determine how similar aneuploid karyotypes of the adapted cultures were in comparison with cancer karyotypes, we analyzed copy number data from The Cancer Genome Atlas (TCGA), MSK-IMPACT, and the Cancer Cell Line Encyclopedia (CCLE) (Zehir et al. 2017; Ghandi et al. 2019; The ICGC/TCGA Pan-Cancer Analysis of Whole Genomes Consortium 2020). Since we chose cell lines based on their lack of CIN and aneuploidy prior to the adaptation to reversine, the cancers that they are derived from (chronic myeloid leukemia and colorectal cancers with microsatellite instability) typically have low rates of aneuploidy (Hoadley et al. 2018). We therefore could not make direct comparisons between the aneuploid chromosomes present in the adapted cancer cell lines and frequent aneuploidies in the corresponding cancer type. However, the hTERT immortalized HME1 cell line is derived from a cell type that can lead to breast cancer. Intriguingly, the only cancer type in TCGA that significantly correlates with both the chromosome gains and losses identified in the reversine-adapted HME1 cells is breast cancer (BRCA) (Supplemental Fig. S5). In addition, we combined the data of all of the reversine-adapted populations from the six cell lines and compared them with the combined data of the different cancer types in the three cancer databases. At all three time points, chromosome gain frequencies between cancers and the adapted cell lines were highly correlated (Fig. 2B; Supplemental Fig. S6). This pattern was largely driven by the high frequency of chromosome 8, 20, 5p, and 1q gains in both data sets. The only chromosome that is frequently gained across many different cancers but rarely in the adapted cell lines is chromosome 7. This difference may reflect the negative correlation between chromosome 7q trisomy and other, even more beneficial aneuploidies under reversine selection as described above (Fig. 1G). These data suggest that our approach reconstituted fundamental chromosome gain patterns that are generally associated with proliferation advantages in cancer patients. Furthermore, the data demonstrate that these advantages are largely independent of the underlying microenvironment, as they can be recapitulated by cell lines in culture. Curiously, the similarity between chromosome losses in the adapted cell lines and cancers was much weaker. This could indicate that the monosomies in the adapted populations are more specific to reversine resistance than general proliferation.

### Specific arm aneuploidies are associated with better growth in reversine

We next wanted to identify those aneuploidies that lead to reversine resistance. We therefore determined which of the recurrent arm aneuploidies in the adapted cell lines best correlated with improved growth in reversine. In addition to measuring growth in reversine, we also calculated growth in reversine relative to growth without the drug to account for potential negative effects on general fitness due to the aneuploidy burden. However, the complexity of the karyotypes at 90 d made the detection of significant correlations challenging. In addition, it was impossible to draw correlations in cases where aneuploidy of a particular chromosome was no longer observed. We therefore focused on looking for correlations with growth under reversine for aneuploidies that were found in 25%–80% of adapted populations at 90 d. For those with a frequency >80%, we looked at the 60-d time point when there were enough disomic populations for meaningful comparisons (Supplemental Fig. S7A,B).

**Figure 3.**
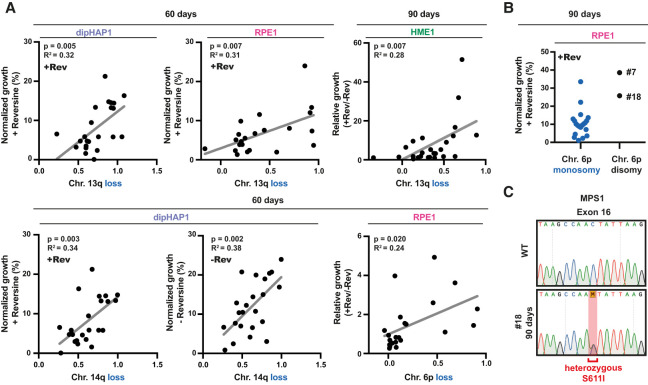
Specific arm aneuploidies correlate with better growth in reversine. (*A*) Scatter plot of adapted populations from dipHAP1, RPE1, and HME1 cells harboring specific chromosome losses (0 indicates no loss, and 1 indicates chromosome loss; *X*-axis) versus their normalized (+Rev or −Rev) or relative (+Rev/−Rev) growth (*Y*-axis). Proliferation from colony formation assays was normalized to the untreated parental cell lines (WT). *n* = 2. Chromosome loss copy number data are from read frequencies using Illumina sequencing. *P*-values are from *F*-tests. (*B*) Normalized growth in reversine of the 90-d adapted RPE1 populations grouped by chromosome 6p copy number state. The two adapted RPE1 populations with 6p disomy are labeled with their cell line IDs (#7 and #18). (*C*) Sanger DNA sequencing chromatogram showing the presence of a heterozygous single-base-pair substitution in exon 16, leading to the *MPS1*^*S611I*^ mutation in RPE1 population #18 at the 90-d time point.

We found positive correlations between reversine growth and 13q monosomy in both RPE1 and dipHAP1 cells at the 60-d time point and in HME1 cells at the 90-d time point (Fig. 3A; Supplemental Fig. S7A,B). In addition, we observed positive correlations with chromosome 6p loss in RPE1 cells and chromosome 14q loss in dipHAP1 cells after 90 d of adaptation. However, the growth improvement of chromosome 14q loss was just as strong in the absence of reversine, suggesting that the selection of this chromosome may not be reversine-specific. Positive correlations were also seen in both DLD1 and HME1 cells with 20q. However, in dipHAP1 cells, the gain of chromosome 20 was negatively correlated with growth despite being present in ∼50% of the adapted populations, emphasizing the difficulties of identifying the contributions of individual chromosomes in complex karyotypes. The two strongest candidates for aneuploidies that lead to a strong reversine resistance are therefore chromosome 6p loss in RPE1 cells and chromosome 13 loss in multiple cell lines.

### Point mutations that cause reversine resistance affect aneuploidy patterns

The correlation between reversine resistance and chromosome 6p monosomy was significant at 60 d of adaptation, yet this trend was reversed at the 90-d time point (Fig. 3B). Surprisingly, after 90 d, the only two adapted RPE1 strains without chromosome 6p monosomy had extremely high reversine resistance. We hypothesized that these cell lines adapted through point mutations instead, decreasing the need for beneficial aneuploidies. Since mutations in *MPS1* had previously been shown to increase reversine resistance (Koch et al. 2016), we sequenced the kinase domain of 10 of the adapted RPE1 cell lines. The only cell line with a mutation in *MPS1* was indeed one of the two that did not obtain chromosome 6p monosomy (Fig. 3C). The identified heterozygous S611I mutation is in the same amino acid that was previously demonstrated to confer resistance to reversine and multiple other MPS1 inhibitors (Koch et al. 2016). Since the *MPS1* gene is not present on chromosome 6p, we conclude that mutations present on one region of the genome can affect aneuploidy patterns in other regions of the genome.

### Adapted cells with specific aneuploidies rescue reversine treatment by increasing the duration of mitosis

**Figure 4.**
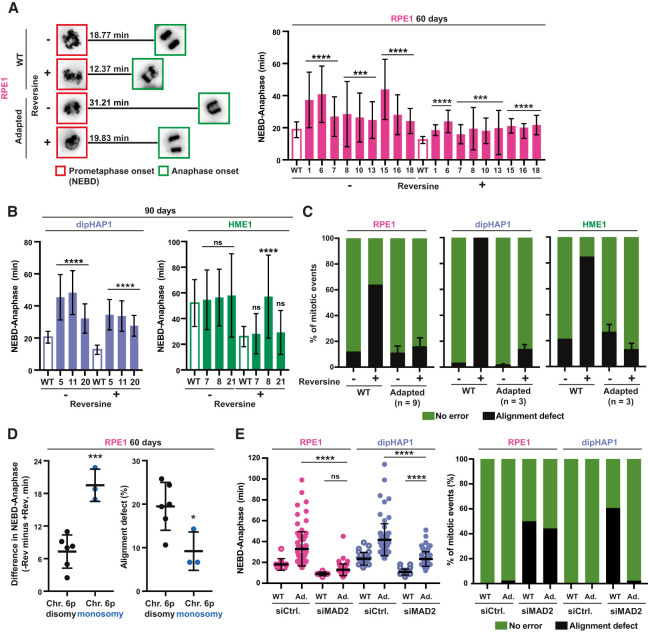
Adapted cells with specific aneuploidies limit CIN by increasing mitotic duration. (*A*, *left*) Representative images from a time lapse of SiR-DNA-stained mitotic RPE1 cells at NEBD (red squares) and at anaphase onset (green squares) (full time-lapse is displayed in Supplemental Fig. S8B). The mean duration in minutes from NEBD to anaphase (*n* > 25 mitoses) is shown. (*Right*) Quantification of time from NEBD to anaphase (in minutes) with and without reversine for the unadapted parental RPE1 cell line (WT; empty bar) and nine adapted RPE1 cell lines (ID numbers; filled bars) at the 60-d time point. *n* = 3. (*B*) Quantification as in *A*, except for dipHAP1 and HME1. Unadapted parental cell lines (WT; empty bar) and three adapted populations (ID numbers; filled bars) each are depicted. *n* = 3. (*C*) Quantification of chromosome misalignment with and without reversine for the unadapted parental cell lines and the adapted cell populations from *A* and *B*. Alignment defects were measured as chromosomes that were not at the metaphase plate in the frame directly preceding anaphase. Error bars depict SD between the adapted populations (*n* depicted in the graph) of the respective cell line. (*D*, *left*) Difference in the NEBD-to-anaphase timing for 60-d adapted RPE1 populations with and without reversine. (*Right*) Percentage of mitoses with alignment defects with reversine in 60-d adapted RPE1 populations. Both are classified by the copy number state of chromosome arm 6p. The cell lines are the same as in *A*. (*E*, *left*) NEBD-to-anaphase duration for parental, 60-d adapted RPE1, and 90-d adapted dipHAP1 cell lines treated with either siRNAs for *MAD2* or a scrambled control (siCtrl.). (Ad.) Adapted. Bars represent mean ± SD. *P*-values are from unpaired Student's *t*-tests. (*) *P* < 0.05, (***) *P* < 0.001, (****) *P* < 0.0001, (ns) not significant.

Next, we investigated whether increased reversine resistance was associated with decreased mitotic errors in the adapted cell lines. For this, we monitored mitotic timing and chromosome segregation fidelity by live-cell imaging of unsynchronized populations stained with SiR-DNA (Fig. 4A,B; Supplemental Fig. S8A,B). The analysis comprised 15 adapted cell populations (nine from RPE1 time point 60 d and three from dipHAP1 and three from HME1 time point 90 d) that showed extensive karyotype diversity in the most commonly acquired aneuploidies: 6p loss, 13q loss, and 20q gain.

Elongation of mitotic duration has been previously proposed as a resistance mechanism to MPS1 inhibition (Sansregret et al. 2017). Although the three parental cell lines had substantial differences in mitotic duration (∼19 min in RPE1 and dipHAP1 cells vs. ∼50 min in HME1 cells), the average duration from nuclear envelope breakdown (NEBD) to anaphase was reduced after reversine addition by ∼6–20 min (Fig. 4A,B; Santaguida et al. 2010). Strikingly, for the adapted cell lines, the mitotic timing in the presence of reversine was now generally more similar to the timing of unadapted cells (WT) in the absence of reversine. This suggests that cells may adapt to reversine by extending the mitotic duration to provide additional time for chromosome alignment. Notably, upon reversine removal, mitotic timing became exceedingly long in adapted cells, with a fraction of mitoses being >120 min (Supplemental Fig. S8C).

The shortened mitotic timing after reversine treatment greatly increases the number of misaligned chromosomes. To determine whether the adapted cells had improved chromosome alignment, we measured the percentage of cells with one or more chromosomes that where not properly aligned at the metaphase plate immediately before anaphase onset. Adapted cells exhibited very low chromosome misalignment rates in the presence or absence of reversine (Fig. 4C). For RPE1 cells, we identified correlations between the duration of mitotic timing with reversine and both the relative growth in reversine and the number of alignment errors (Supplemental Fig. S8D,F). In addition to misaligned chromosomes, reversine addition also substantially increases the formation of lagging chromosomes and chromosome bridges in RPE1 and dipHAP1 cells. The adapted cell lines display a decrease in the rates of anaphase errors in the presence of reversine (Supplemental Fig. S8E). These results indicate that the adapted cells limit reversine-induced mitotic error rates by extending mitotic duration.

After having determined that the adapted cell lines display an increased mitotic duration and decreased CIN in reversine, we next asked whether these traits were associated with any recurrent aneuploidies. We found that loss of chromosome 6p, which is associated with better relative growth in reversine (Fig. 3A; Supplemental Fig. S7B), is also associated with increased mitotic length and decreased chromosome misalignment rates (Fig. 4D).

Since MPS1 activates the SAC and we used reversine concentrations that do not completely inhibit its activity, cells could adapt either by increasing the residual SAC activity or by inhibiting activities downstream from the SAC such as the APC/C. We hypothesized that in reversine-adapted cells, SAC and/or APC/C activity could be altered to partially restore mitotic timing in the presence of the drug. To test this, we used small interfering RNA (siRNA) to deplete the essential SAC component MAD2 in RPE1 and dipHAP1 parental and adapted cell lines (Supplemental Fig. S9A). Similar to reversine treatment, MAD2 depletion in unadapted cells led to a decrease in mitotic length and increased alignment errors (Fig. 4E; Supplemental Fig. S9B). MAD2 depletion eliminated the mitotic delay phenotype exhibited by adapted RPE1 cells in the absence of reversine. Upon SAC inactivation, both unadapted and adapted RPE1 cells became extremely similar in mitotic duration and chromosome misalignment rates. These results indicate that in adapted RPE1 cells, the activity of the SAC is modulated to counteract the effects of partial MPS1 inhibition. In contrast to RPE1 cells, mitotic length in dipHAP1 cells was still significantly higher than in unadapted cells after MAD2 depletion. In addition, MAD2 depletion in the adapted dipHAP1 cells did not increase chromosome misalignment rates. These partial phenotypes could be due to either dipHAP1 cells adapting through mechanisms downstream from the SAC or only partial inhibition of SAC activity by the MAD2 depletion. However, nocodazole treatment led to a fully penetrant mitotic arrest in control cells and caused mitotic slippage in 100% of MAD2-depleted RPE1 and dipHAP1 cells, indicating robust SAC inactivation (Supplemental Fig. S9B). We conclude that RPE1 cells adapt to reversine primarily by promoting SAC activity, while dipHAP1 cells adapt through a combination of increased SAC activity and additional effects downstream from the SAC such as the APC/C.

### Engineered individual monosomies are sufficient for partial reversine resistance

Next, we sought to identify the basis behind the frequent selection of specific aneuploidies in the adapted cell lines. We focused on the most common chromosome losses, as we were able to efficiently engineer specific chromosome and gene losses as opposed to gains. We chose chromosome arm 13q loss, since it is one of the most frequently selected aneuploidies across all reversine-adapted cell lines (Fig. 2A). In addition, we decided to examine monosomy of chromosome 6p, since it correlated well with improved reversine resistance in RPE1 cells (Fig. 3A; Supplemental Fig. S7B). To ascertain the specific roles of these monosomies in reversine resistance, we engineered the losses of chromosome arms 6p and 13q individually in the dipHAP1 cell line with CRISPR (Supplemental Fig. S10A).

**Figure 5.**
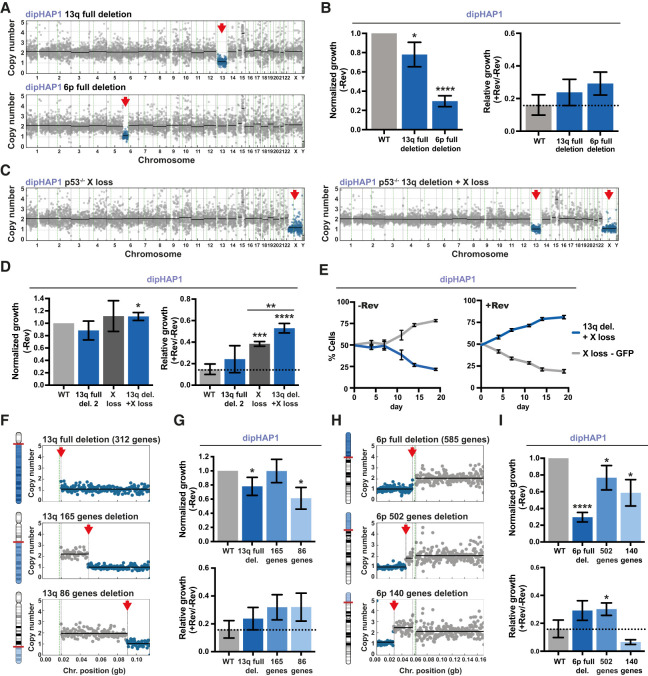
Engineered individual monosomies and partial chromosome deletions can recapitulate reversine resistance. (*A*) Copy number profiles of engineered dipHAP1 cell lines with the deletion of chromosome arm 13q or 6p. Blue dots represent the region of chromosome loss, which is highlighted with a red arrow. (*B*) Normalized (−Rev) and relative (+Rev/−Rev) growth of the depicted 13q and 6p deletion cell lines. Proliferation measurements are from colony formation assays. Normalized growth (−Rev) represents the growth of the cell line without reversine normalized to the proliferation of the dipHAP1 parental cell line (WT) without reversine. The relative growth (+Rev/−Rev) represents the proliferation of the cell line with reversine (+Rev) relative to its growth without reversine (−Rev). The dotted line depicts mean relative growth of dipHAP1 WT cells with reversine. *n* = 3. (*C*) Copy number profiles of engineered dipHAP1 cell lines with the loss of chromosome X and chromosome 13q deletion in addition to X. (*D*) Normalized and relative growth of the depicted 13q, X, or double-monosomic cell line. For WT and 13q deletion 2, *n* = 6; for X loss and 13q deletion + X loss, *n* = 3. (*E*) Cocultivation of a GFP-labeled cell line with the monosomy of chromosome X and a cell line with monosomies of both X and 13q with and without reversine. The mean ± SD for six separate populations of cells in one time course is shown. (*F*) Representative depiction and chromosome-specific copy number profiles of engineered partial deletions of chromosome arm 13q. (*G*) Normalized and relative growth of the 13q partial deletion cell lines. *n* = 3. (*H*) Representative depiction and chromosome-specific copy number profiles of engineered partial deletions of chromosome arm 6p. (*I*) Normalized and relative growth of the 6p partial deletion cell lines. *n* = 3. Bars depict mean ± SD. *P*-values are from unpaired Student's *t*-tests. (*) *P* < 0.05, (**) *P* < 0.01, (***) *P* < 0.001, (****) *P* < 0.0001. Where not indicated, the *P*-value was >0.05.

To generate cell lines with one specific chromosome arm loss, we selected sgRNAs that target the chromosome arm either at one site close to the centromere or at a repetitive region in the chromosome arm (Supplemental Fig. S10B; Supplemental Table S3; Zuo et al. 2017). Two different sgRNAs for each chromosome arm deletion were used to account for off-target effects of the individual sgRNAs. Using this approach, we successfully engineered two 13q monosomy and three 6p monosomy cell lines in dipHAP1 cells. These cell lines contained no other CNAs (Fig. 5A; Supplemental Fig. S10C). Attempts to engineer chromosome losses in RPE1 cells were unsuccessful, potentially due to differences in DNA repair mechanisms.

In the absence of reversine, engineered dipHAP1 cells monosomic for chromosome arm 13q showed a mild growth reduction, whereas deletion of one copy of 6p resulted in very poor growth (Fig. 5B; Supplemental Fig. S10D,E). Both monosomies showed a better growth in reversine relative to its absence when compared with wild-type dipHAP1 cells as measured by colony formation assays. However, for the 6p deletion, the added reversine resistance could not overcome the negative effect of the aneuploidy on proliferation, which may explain why this CNA was not observed in the adapted dipHAP1 cells.

Occasionally, other aneuploidies were observed during the screening process for 6p and 13q loss. These include cell lines whose only CNA is the loss of chromosomes X, Xp, and 19p (Supplemental Fig. S10F). As mentioned earlier, the loss of chromosome X results in a relief from aneuploidy in dipHAP1 cells and was observed in all of the reversine-adapted populations (Supplemental Fig. S4C). Similar to 6p and 13q monosomy, the loss of the whole X chromosome showed an increased reversine resistance compared with euploid wild-type cells (Supplemental Fig. S10G). Loss of only the p-arm of X did not show this phenotype, suggesting that the genes affecting reversine resistance are on the q-arm. Loss of 19p was not observed in any of the adapted cell lines. We therefore used this aneuploidy as a control for the general effects of chromosome loss. Chromosome 19p loss made the cells more sensitive to reversine, demonstrating that reversine resistance is not a general property of monosomies (Supplemental Fig. S10H). We conclude that the loss of one copy of chromosomes 6p, 13q, or X is sufficient to confer reversine resistance.

Notably, the rescue phenotypes for the engineered monosomies were much weaker than what was observed in the adapted cell lines, suggesting that multiple aneuploidies act cooperatively during the adaptation process. To test whether multiple monosomies can act together to create stronger drug resistance phenotypes, we used a chromosome 13q deletion cell line that also acquired chromosome X loss during the generation of the cell line (Fig. 5C). The combined double-monosomic cell line had substantially greater reversine resistance than either monosomy individually as measured using colony formation assays (Fig. 5D). To additionally measure reversine resistance with a more sensitive assay, we performed a cocultivation assay between a GFP-labeled cell line with chromosome X monosomy and an unlabeled cell line with chromosome X and chromosome 13q monosomy (Supplemental Fig. S11A–D). In the absence of reversine, the cell line with only a single aneuploidy grew better, but with the addition of reversine, the double-monosomic cell line quickly and reproducibly took over the population (Fig. 5E; Supplemental Fig. S11C). These results demonstrate that multiple monosomic chromosomes can create stronger phenotypes and support the idea that the reversine resistance in the adapted cell lines results from the additive or synergistic contributions of multiple aneuploid chromosomes.

### Identification of chromosomal regions that are sufficient to confer reversine resistance

The losses of chromosome arms 6p and 13q decrease the copy number of hundreds of genes on the aneuploid chromosome. To narrow down the chromosomal regions that confer drug resistance, we engineered partial deletions of the chromosome arms. We engineered two partial deletions that eliminate either 165 or 86 of the 312 genes on chromosome 13q (Fig. 5F; Supplemental Fig. S12A). Both of these deletions exhibited robust reversine resistance that was similar to the full arm deletion (Fig. 5G; Supplemental Fig. S12B). This indicates that either one or more of the 86 genes closest to the telomere contribute to reversine resistance when heterozygously deleted or another genetic element in this region is responsible. In addition to full arm loss, we also detected focal losses on chromosome arm 13q in the adapted dipHAP1 and HCT116 cell lines (Supplemental Fig. S12C). Focal losses of chromosome arm 13q in the adapted cell lines always contained our identified chromosomal region, further supporting the results from the engineered partial deletions.

The full deletion of chromosome arm 6p decreases the copy number of 585 genes. We again made two partial deletions—one that eliminated 502 genes and another that only removed 140 genes (Fig. 5H; Supplemental Fig. S12D). Interestingly, although both of the smaller deletions grew better in the absence of reversine than the full arm deletion, only the larger 502 gene deletion still showed better growth in reversine than without reversine relative to the parental cell line (Fig. 5I; Supplemental Fig. S12E). This narrows down the region of interest to the one in between the partial deletion sites containing 362 genes. Importantly, these results further demonstrate that the reversine resistance is due to the loss of specific regions and not a general effect of aneuploidy or the technique used for aneuploidy generation.

### Identification of genes that confer reversine resistance when heterozygously deleted

**Figure 6.**
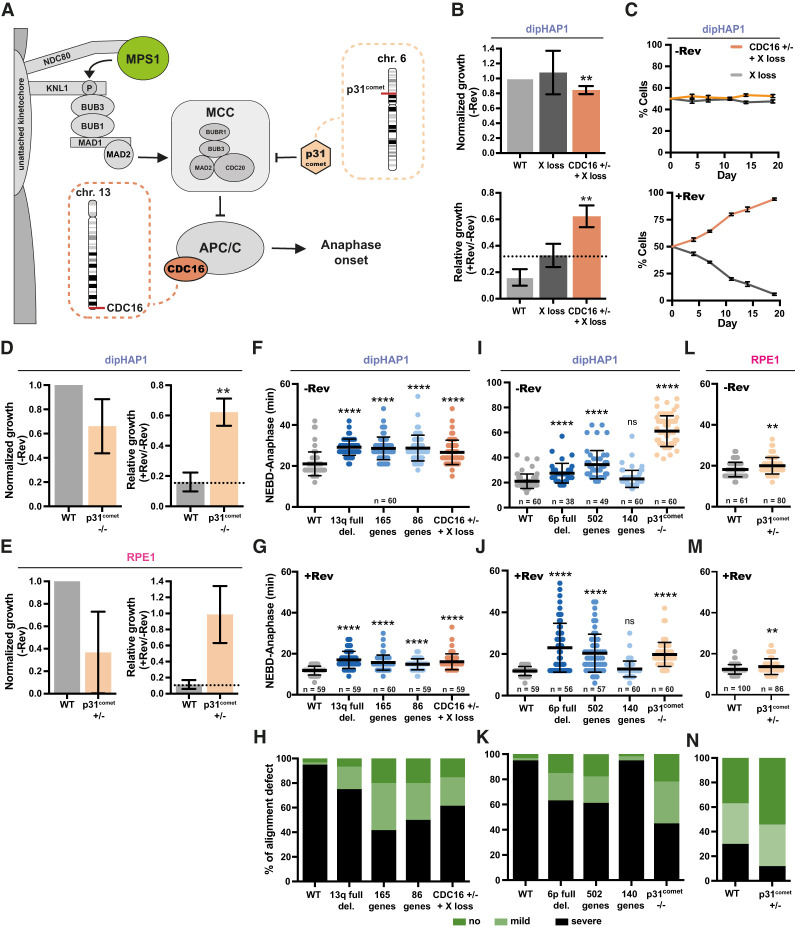
Identification of the cellular mechanism underlying reversine resistance. (*A*) Simplified model of the SAC and the roles of CDC16 and p31^comet^. (*B*) Normalized and relative growth of the dipHAP1 *CDC16* heterozygous knockout (+/−, +X loss) and an X loss cell line as control. Proliferation measurements are from colony formation assays. Normalized growth (−Rev) represents the growth of the cell line without reversine normalized to the proliferation of the dipHAP1 parental cell line (WT) without reversine. The relative growth (+Rev/−Rev) represents the proliferation of the cell line with reversine (+Rev) relative to its growth without reversine (−Rev). The dotted line represents mean relative growth of the X loss cell line. *n* = 3. (*C*) Cocultivation of the GFP-labeled X loss cell line and a cell line with *CDC16* heterozygous knockout and monosomy of chromosome X with and without reversine. The mean ± SD for six separate populations of cells in one time course is shown. (*D*) Normalized and relative growth of the parental dipHAP1 and *p31*^*comet*^ homozygous knockout cell lines. *n* = 2. (*E*) Normalized and relative growth of the parental RPE1 and *p31*^*comet*^ heterozygous knockout cell lines. *n* = 2. (*F*,*G*) Duration from NEBD to anaphase in minutes for the engineered dipHAP1 13q deletions and the *CDC16*^+/−^ knockout cell line without reversine (−Rev; *n* = 60 mitoses) (*F*) and with reversine (+Rev; *n* = 59–60 mitoses) (*G*). (*H*) Quantification of no (dark green), mild (one unaligned chromosome; light green), or severe (multiple unaligned chromosomes; black) alignment defects of the mitoses from the dipHAP1 parental, 13q partial deletion, and *CDC16*^+/−^ knockout cell lines. (*I*,*J*) Duration from NEBD to anaphase in minutes for the engineered dipHAP1 6p deletions and the *p31*^*comet*^^−/−^ knockout cell line without reversine (−Rev; *n* = 38–60 mitoses) (*I*) and with reversine (+Rev; *n* = 56–60 mitoses) (*J*). SiR-DNA staining efficiency was increased by adding 10 µM verapamil. (*K*) Quantification of the alignment defect of the measured mitoses from the parental, 6p partial deletion, and *p31*^*comet*^^−/−^ knockout cell lines as in *H*. (*L*,*M*) Duration from NEBD to anaphase in minutes for the RPE1 *p31*^*comet*^^+/−^ knockout cell line without reversine (−Rev; *n* = 61–80 mitoses) (*L*) and with reversine (+Rev; *n* = 86–100 mitoses) (*M*). (*N*) Quantification of the alignment defect of the parental RPE and *p31*^*comet*^^+/−^ knockout cell lines as in *H* and *K*. For *F*–*K*, *n* = 3; for *L*–*N*, *n* = 5. Bars depict mean ± SD. *P*-values are from unpaired Student's *t*-tests. (**) *P* < 0.01, (****) *P* < 0.0001, (ns) not significant. Where not indicated, the *P*-value was >0.05.

Monosomy of chromosomes can induce phenotypes either through loss of heterozygosity (LOH) or decreased expression of genes on the chromosome. Since the monosomies were engineered in the dipHAP1 cell line that is already homozygous for nearly the entire genome (with the exception of a portion of chromosome 15), we can eliminate LOH as a possible contributor to the aneuploidy phenotypes. After further refining the regions of interest on chromosomes 13q and 6p, we took a candidate approach to identify genes that may contribute to the phenotype. The 86-gene region on 13q contains the gene for *CDC16*, an essential component of the APC/C (Fig. 6A). A mutation in *CDC16* was previously identified as conferring reversine resistance, making it a strong candidate for a gene that could contribute to resistance when heterozygously deleted (Sansregret et al. 2017). We therefore used CRISPR to engineer a heterozygous deletion of *CDC16* in dipHAP1 cells (Supplemental Fig. S13A). Whole-genome sequencing of the identified cell line revealed that it also contained a deletion of chromosome X (Supplemental Fig. S13B). We therefore used the chromosome X deletion cell line described above as the control for experiments with this cell line. *CDC16*^+/−^ cells showed a strong resistance to reversine, substantially greater than the resistance observed for X loss alone (Fig. 6B). CDC16 Western blot analysis of the deletion and knockout cell lines further revealed that heterozygous deletions can impact protein levels (Supplemental Fig. S13C). Additionally, cocultivating the *CDC16* knockout cell line with a GFP-labeled X loss control cell line showed that the knockout cell line quickly overtook the population in medium containing reversine in two independent experiments (Fig. 6C; Supplemental Fig. S13D–G). This level of resistance is consistent with the *CDC16* gene being primarily responsible for the reversine resistance in the chromosome 13q deletion cell lines, indicating that the loss of CDC16 is likely the driving force behind the selection of chromosome 13q monosomy in the adapted cell lines.

The 362-gene region on chromosome 6p contains two genes of primary interest for potentially contributing to reversine resistance: *p31*^*comet*^ and *p21^CDKN1A^.* p31^comet^ is a negative regulator of the mitotic checkpoint complex (MCC) that was previously identified in a CRISPR/Cas9 screen for MPS1 resistance (Fig. 6A; Thu et al. 2018). *p21*^*CDKN1A*^ is a tumor suppressor gene that promotes cell cycle arrest following chromosome missegregation (Karimian et al. 2016). Although p21^CDKN1A^ frequently acts through p53, it also has p53-independent functions that might contribute to reversine resistance in our *p53*-deleted cell lines (Matsuda et al. 2017). However, neither heterozygous nor homozygous deletion of *p21*^*CDKN1A*^ conferred reversine resistance in dipHAP1 cells (Supplemental Fig. S14A–C). For *p31*^*comet*^, we obtained a homozygous deletion cell line in dipHAP1 cells and a heterozygous deletion cell line in RPE1 cells (Supplemental Fig. S14D,E). Both cell lines showed strong resistance to reversine (Fig. 6D,E). p31^comet^ protein abundance was proportional to the gene copy in the engineered cell lines (Supplemental Fig. S14F). We conclude that the heterozygous deletion of *p31*^*comet*^ is sufficient for robust reversine resistance and that the decrease in p31^comet^ expression is likely the principal basis behind the selection of 6p loss in RPE1 cells adapted to reversine.

### Characterization of the cellular mechanisms behind the reversine resistance

The adapted dipHAP1 and RPE1 cell lines showed an increased time in mitosis compared with the parental cell lines (Fig. 4A,B). In addition, they displayed improved chromosome alignment in the presence of reversine (Fig. 4C). We next determined whether the engineered monosomies and heterozygous knockout cell lines that induce reversine resistance also rescue the cell cycle timing and chromosome alignment defects.

The chromosome 13q monosomy, the partial chromosome 13 deletions, and the *CDC16* heterozygous deletion all had nearly identical NEBD-to-anaphase delays of ∼8 min in the absence of reversine (Fig. 6F). When reversine was added, the mitotic timing was reduced for all of the mutants yet still significantly higher than for the parental cell line (Fig. 6G). Similarly, all of the cell lines affecting chromosome 13 partially rescued the reversine-induced chromosome alignment defect (Fig. 6H). The chromosome 6p full and the 502-gene deletions also had a highly significant increase in mitotic duration both with and without reversine and a rescue of chromosome alignment (Fig. 6I–K). In addition, the *p31*^*comet*^ homozygous deletion in dipHAP1 cells resulted in extremely long mitotic delays with and without reversine and a robust rescue of the alignment defects. In contrast, the 140-gene partial deletion that did not rescue reversine resistance did not show an anaphase delay or a rescue of chromosome alignment. In RPE1 cells, the *p31*^*comet*^^+/−^ cell line had a small but significant anaphase delay in both the presence and absence of reversine (Fig. 6L,M). An improvement in chromosome alignment was also observed in this cell line (Fig. 6N). As with the rescue of proliferation in reversine, these phenotypes were not as strong as what was observed for the adapted cell lines, further suggesting that the adaptation is due to the cumulative effects of multiple genomic changes.

To determine whether the heterozygous loss of *CDC16* and *p31*^*comet*^ is not just sufficient but also necessary for the observed mitotic delays, we increased the expression of these genes in the engineered chromosomes 13 and 6p monosomies, respectively. Gene expression in dipHAP1 cells was amplified with the CRISPRa system (Perez-Pinera et al. 2013; Chavez et al. 2015) using single guide RNAs targeted to the promoters of the genes that resulted in a 2.5-fold to 3.5-fold increase in mRNA levels (Supplemental Fig. S15A–E). Addition of the guide RNAs eliminated the mitotic delay for the *CDC16* heterozygous deletion and chromosome 6p monosomic cell lines with and without reversine. For chromosome 13 monosomy, mitotic timing was substantially, but not completely, reduced to the levels of the empty vector, indicating that genes other than *CDC16* on this chromosome also contribute slightly to the anaphase delay.

A recent study suggests that the presence of monosomic chromosomes generally increases mitotic duration in RPE1 cells (Chunduri et al. 2021). To determine whether the elongation of mitosis is a general trait of our engineered monosomies, we once again used the dipHAP1 cell line with the loss of chromosome 19p (591 genes). This cell line showed no differences in mitotic timing with or without reversine and no rescue of the chromosome alignment defects (Supplemental Fig. S15F,G). We conclude that the mitotic delay is not caused by monosomy in general in dipHAP1 cells and that the observed phenotypes are chromosome-dependent. Overall, these results are consistent with the reversine growth data, demonstrating a clear link between mitotic timing, chromosome alignment, and drug resistance for the engineered aneuploid and gene knockout cell lines.

## Discussion

In this study, we developed a time-resolved adaptation assay for human cells to study complex karyotype formation over time in response to long-term MPS1 inhibition in six different cell lines. Reversine-mediated inactivation of the SAC leads to high rates of CIN that also serve as a selective force to adapt via aneuploidy (Fig. 1A). Thus, our strategy is comparable with one that we previously used to generate complex aneuploidy in yeast (Ravichandran et al. 2018). Similar to what was observed in yeast, independently cultivated populations became more homogenous over time and converged on optimal karyotypes.

During the development of these optimized karyotypes, we observed different types of patterns that bear a striking similarity to those previously reported in cancer cells. Some of the most frequently obtained aneuploidies were observed across many of the adapted cell lines. This is analogous to certain aneuploidies being frequently observed in the majority of cancer types such as the gain of chromosome 8 or 20. In contrast, other aneuploidies in the adapted cell lines were only observed in a single cell line. These included 6p loss in RPE1 and 16q loss in HME1. Such cell line-specific aneuploidy patterns reflect cancer type-specific aneuploidies, which include chromosome 16 loss in breast and ovarian cancers and chromosome 3p gain specifically in sarcomas (Hoadley et al. 2018). This cell line specificity could be due to either differential gene expression, interactions with other common mutations, or both. In support of the second hypothesis, we found a mutation in the *MPS1* gene specifically in a cell line with a karyotype that was highly divergent from the other adapted populations. Altered aneuploid karyotypes resulting from specific mutations have also recently been reported in yeast (Clarke et al. 2022). In conclusion, the ability to recapitulate both general and cell line-specific aneuploidy patterns in human cells is an important step in determining the bases behind these patterns. Moreover, we found a strong correlation between chromosome gain patterns observed in our CIN-adapted cell populations and those frequently observed in cancer, including the gains of chromosomes 1q, 5p, 8, and 20. This suggests that the conditions for acquiring chromosome gains are independent of microenvironment and instead reflect more general cell-autonomous proliferation advantages through alterations in the cell cycle machinery. This result is in agreement with the proposed roles for these aneuploidies in promoting proliferation through the up-regulation of genes involved in cell cycle entry and DNA repair (Scotto et al. 2008; Tabach et al. 2011; Dehner et al. 2021; Su et al. 2021).

In addition, we observed that smaller CNAs become more prevalent over time, with an increase in arm-level and segmental copy number changes relative to whole-chromosome aneuploidies. Since the benefits of aneuploidy come at the cost of expression imbalances of the other genes present on the aneuploid chromosome, reducing the number of imbalanced genes would be advantageous. The increase in arm aneuploidy frequencies over time has also been observed in cancer karyotype analyses (Shukla et al. 2020). Our observations suggest a basis for this trend, in which whole-chromosome aneuploidies are converted to arm-level or smaller CNAs over time within a population to partially relieve the aneuploidy burden.

Furthermore, we observed strong negative correlations between specific aneuploid chromosomes. Such correlations are also observed in cancer karyotypes (Ravichandran et al. 2018; Shukla et al. 2020). Over time, the more beneficial aneuploidy replaces the less beneficial one, such as with the transition from chromosome 7 trisomy to chromosome 14 monosomy in dipHAP1 cells. Importantly, this type of negative pattern would not be observable by simply looking at the karyotypes at later time points, such as in fully developed cancers. The replacement of one aneuploid chromosome with another may also provide the basis behind the frequent chromosome-level copy number-neutral loss of heterozygosity in cancer (Nichols et al. 2020; Ciani et al. 2022). In yeast, these negative correlations can result from genetic interactions between the aneuploid chromosomes, which may also be the basis behind them in human cells (Ravichandran et al. 2018).

The final similarity between the aneuploidy patterns we observed and cancer karyotypes is that the HCT116 and DLD1 colorectal cell lines used in this study that exhibit microsatellite instability (MSI) acquire very few chromosome gains and no chromosome losses. This observation is in line with the mutual exclusivity between MSI and CIN in colorectal cancers (Lengauer et al. 1997; Eshleman et al. 1998). The complete lack of chromosome losses in these cell lines may reflect the accumulation of mutations that recapitulate loss-of-function phenotypes similar to chromosome losses. In contrast, the increased expression caused by chromosome gains may be more difficult to obtain via mutations acquired from a loss of mismatch repair, which may explain why the DLD1 cell line still acquires chromosome gains. Overall, the ability to recapitulate these types of aneuploidy patterns in human cells through adaptation experiments gives insights into the bases behind their formation and provides a handle for directly testing the driving forces behind many of the most prominent motifs observed in cancer karyotypes.

Correlations between growth in reversine and specific CNAs in the adapted cell lines implicated 13q loss in multiple cell lines and 6p loss in RPE1 cells with resistance to the drug. In addition, chromosome 6p monosomy was correlated with increased mitotic timing and improved chromosome alignment in the presence of reversine. Engineered monosomies of chromosome arms 13q and 6p recapitulated reversine resistance, extended mitotic timing, and decreased mitotic errors. Down-regulation of p31^comet^ or CDC16 through the monosomies of chromosome arms 6p or 13q should both lead to decreased APC/C activity, which would delay anaphase onset. It has previously been shown that the deletion of the APC/C subunits *APC7* or *APC16*, which also should lead to a decreased APC/C activity, rescues the lethality of *MAD2* deletion and therefore SAC inactivation in HCT116 cells (Wild et al. 2018). Interestingly, truncation mutations in the APC/C subunit *CDC27* are frequently found in cancer, and cell lines engineered with these mutations display elongated mitotic timing and reduction of CIN (Sansregret et al. 2017). In agreement with these previous results, heterozygous deletions of either *p31*^*comet*^ or *CDC16* in dipHAP1 cells showed resistance to SAC inhibition and extended mitotic timing and were sufficient to reduce CIN. This indicates that the copy number changes of these genes were the drivers of the acquisition of the monosomies of chromosome arms 13q and 6p in the adapted cell lines. In general, our results demonstrate that specific aneuploidies can have impactful effects on cell cycle timing. How widespread this is in cancer and whether other stages of the cell cycle are also affected by specific aneuploidies is currently unknown.

Attempts to recapitulate cancer aneuploidy phenotypes in human cells and determine the genetic basis behind them have been extremely challenging (Ben-David and Amon 2020). Recently, there have been a few breakthroughs in identifying and understanding the role of individual frequent aneuploidies in human cells. For instance, resistance to the microtubule-stabilizing drug paclitaxel has been demonstrated via the acquisition of the loss of chromosome 10 in RPE1 cells (Lukow et al. 2021). In addition, a series of experiments using mouse and human cell models for Ewing sarcoma associated the gain of chromosome 8 with the up-regulation of the *RAD21* and *MYC* genes that reside on that chromosome (Su et al. 2021). Despite the challenges in determining the basis behind aneuploidy selection in human cells, there are multiple examples from studying aneuploidy in yeast (Selmecki et al. 2006; Rancati et al. 2008; Chen et al. 2012, 2015; Ravichandran et al. 2018). In these cases, the benefits of aneuploidy under selective conditions can often be attributed to only one or two genes. Whether this also holds true for the much larger human chromosomes was unknown.

Here, we identified two aneuploidies that were sufficient for a relative increase in resistance to the drug reversine and determined that a single gene on each chromosome is sufficient to recapitulate the phenotype when heterozygously deleted, both qualitatively and quantitatively. Our results suggest that single genes can provide the selective advantage behind the misregulation of hundreds of genes on a human chromosome. This result is in contrast to computational models of aneuploidy patterns in cancer that attribute the selection of chromosome gains and losses to multiple oncogenes and tumor suppressors across the length of a chromosome (Davoli et al. 2013; Sack et al. 2018). However, these differences between in silico models and in vivo models could be attributed to the differences in the diverse selective forces at play during tumorigenesis and the strong, specific selection from a small molecule inhibitor. Determining the basis behind the gains of chromosomes 5, 8, and 20 that are both common in cancer and observed in our adaptation experiments will help resolve this discrepancy. Furthermore, the contribution of single genes to aneuploidy selection could be more applicable to other processes such as chemotherapy resistance.

## Materials and methods

### Cell lines and cell culture

All cell lines used in this study tested negative for mycoplasma contamination. Their tissue types, sources, authentication, and culture conditions are summarized in Supplemental Table S1. All cell lines were cultured in a humidified growth chamber at 37°C and 5% CO_2_ in the respective media. The parental hTERT-RPE1 cell lines with inducible Cas9 (wild type and inducible single-knockout *p53*^−/−^) were kindly provided by I.M. Cheeseman (University of California). The parental HCT116 cell lines (wild type and *p53*^−/−^) were gifts from B. Vogelstein (The Johns Hopkins Oncology Center). DLD1 was provided by M. Baccarini (Max Perutz Laboratories, Vienna). The parental hTERT-HME1 cell line was obtained from Evercyte (CHT-044-0236), and the parental EEB is a cellosaurus cell line (Riken RCB2345). The parental HAP1 cell line was a gift from Joanna Loizou (Research Center for Molecular Medicine of the Austrian Academy of Sciences, Austria).

### Generation of knockout cell lines

*TP53* knockout: To generate the RPE1 *TP53* single knockouts, Cas9 was induced by adding 1 µg/mL doxycycline hyclate (Sigma) every 24 h for 3 d before single-cell sorting. To generate the *TP53* single knockouts in HME1, EEB, and DLD1, a CRISPR/Cas9 was provided on a plasmid (Ran et al. 2013). sgRNA against exon 2 of the *TP53* gene was cloned into pSpCas9(BB)-2A-GFP (PX458; a gift from Feng Zhang; Addgene plasmid 48138). The sgRNA plasmid was transfected into cells using FuGENE HD (Promega). Two days after transfection, cells were sorted for the presence of Cas9 (GFP-positive), and another 3 d later, single-cell sorting into 96-well plates was performed. Clonal populations were expanded gradually over the course of 3 wk. *TP53* mutations were identified by Sanger sequencing, and karyotypes of the cell lines were validated by whole-genome sequencing. *p21*, *p31*^*comet*^, and *CDC16* knockouts: The knockouts in dipHAP1 and RPE1 cells were generated similar to the protocol above, except XtremeGene 9 (Roche) was used as the transfection reagent for dipHAP1 cells and electroporation for RPE1 cells. All sgRNAs used for the generation of knockout cell lines are listed in Supplemental Table S5. The respective genotyping primers are listed in Supplemental Table S6.

### Reversine adaptation assay

To initiate the adaptation process, parental *p53*^−/−^ cell lines were split into 12 independent populations at day 0, and reversine (Axon Medchem BV) was added at the indicated concentrations for the respective cell lines where ∼90% of cells died (Supplemental Fig. S1A). Three days later, single-cell sorting into 96-well plates was performed, and individualized cells were allowed to grow into colonies in the presence of reversine for the next 10–14 d. Around 20 independent cell populations for each cell line were expanded and continuously cultivated in medium containing reversine for up to 90 d. During this time period, the drug was replenished every 3–4 d. Reversine-adapted populations were analyzed at three time points—after 30, 60, and 90 d. For the last 30 d, reversine concentrations were increased to levels that are lethal for unadapted cells to further increase the selection of optimized adapted populations at 90 d (Supplemental Fig. S1B).

### Next-generation sequencing (NGS) and data analysis

Genomic DNA from subconfluent populations was isolated using the QIAamp UCP DNA micro kit (Quiagen). NGS sample preparation was then performed as described previously in Ravichandran et al. (2018). In brief, DNA samples were sheared to ∼500-bp fragments using the Bioruptor Pico sonicator (Diagenode) for two to three cycles (30 sec on/off). Fragmentation efficiency was monitored on a 0.8% agarose gel stained with 1 µM SYTOX green (Thermo Fisher). DNA libraries were prepared with the NEBNext Ultra II DNA library kit for Illumina (NEB). DNA fragments with the optimal size were selected using AMPure XP beads (Beckman Coulter). Up to 96 cell lines per run were barcoded and multiplexed with NEBNext multiplex oligos (index primers 96-well format; NEB) and mixed at equimolar ratios. The multiplexed samples were sequenced using the Illumina HiSeqV4 SR50 setting on an Illumina HiSeq 2500 system and using the Illumina NextSeq2000 P2 SR100 setting on an Illumina NextSeq 2000 system at the Vienna Biocenter Next-Generation Sequencing Facility. All sequencing files are available on the Sequence Read Archive (BioProject ID: PRJNA885752). The demultiplexed data sets were then aligned to the human genome (assembly: GRCh38.p12) using Bowtie2 (version 2.2.9; http://bowtie-bio.sourceforge.net/bowtie2; Langmead and Salzberg 2012), converted to bed files using SAMtools (version 1.3.1; http://samtools.sourceforge.net; Li et al. 2009; Li 2011) and Bedtools (version 2.14; http://bedtools.readthedocs.io; Quinlan and Hall 2010). The resulting bed files were processed through the Gingko cloud software (Garvin et al. 2015) to correct for GC bias in low-read data sets and then analyzed with custom-made Python scripts.

### In-depth analysis of chromosome copy number changes

In-depth characterization of chromosome copy number changes was carried out in three steps: (1) segmentation and change point detection, (2) assignment of segments to corresponding wild-type segments and determination of change state, and (3) correction of oversegmentation.

First, segmentation and change point detection were performed on copy number data that had been GC-debiased before using the Gingko online tool. The array of binned copy number data points (each point representing 500 kb) was first split into segments of equal mean copy numbers that did not contain change points (locations where the mean copy number abruptly changed). By definition, this can be a whole chromosome (no change point), a chromosome arm (one change point at the centromere and none within an arm), or a focal segment (at least one change point within a chromosome arm). The algorithm used to detect change points was PELT (pruned exact linear time) (Killick et al. 2012). By convention, if the algorithm found a change point within a chromosome, it was split additionally at the centromere to distinguish focal from arm copy number changes. Furthermore, for acrocentric chromosomes, only the data of q-arms was processed. The output after change point detection was a sorted list of segments.

For accurate determination of the change state of a segment, a one-to-one assignment between a sample and its corresponding wild-type cell line was implemented. For this, the difference between the mean copy number values of the sample segments and the wild-type segments was measured. Changes of the mean copy number value between sample and wild type >0.5 were marked as gain, and changes less than −0.5 were marked as loss. By convention, changed segments needed to be at least 15 data points (=7.5 Mb) long to get counted. If a change point was found in the sample but not the wild-type or vice versa, an additional change point to ensure the one-to-one correspondence was introduced. For analyses that ignored pre-existing CNAs, certain segments were marked in the wild-type cell lines and excluded from further analysis. If a segment was flagged, all corresponding segments in the sample cell lines were also automatically flagged. The output of this analysis step was a list of segments, labeled with the change state (E for equal or no change, G for gain, and L for loss). Flagged segments were labeled with the state i (ignore). The alignment step described above had the disadvantage of oversegmentation of chromosome data, since it copied change points from wild-type data to the sample data (and vice versa) if the change point was present only in the wild type. A second source of oversegmentation was the heuristic to split automatically at the centromere if at least one change point was found. For correction of this oversegmentation, subsegments of the same change state were merged.

### Colony formation assay

On day 0, 600 cells were seeded into six-well plates and incubated for 12 d in their respective culture media supplemented with reversine at the indicated concentrations (+Rev) or with the corresponding amount of DMSO (−Rev). The following reversine concentrations were used for the adapted cell lines: 400 nM for dipHAP1, 50 nM for DLD1, 100 nM for HCT116, 125 for nM RPE1, and 100 nM for HME1 (Supplemental Fig. S1A). For the engineered dipHAP1 cell lines, 300 nM reversine was used, and for the engineered RPE1 cell lines, 125 nM reversine was used. Colonies were fixed with 4% (v/v) formaldehyde in PBS (Thermo Fisher Scienitific) for 20 min, washed with purified water or PBS, stained for 20–30 min with Crystal Violet, washed with purified water several times, and dried. The plates were imaged using a ChemiDocMP imaging system (Bio-Rad), and analysis was done in ImageJ. The images were thresholded, and the fraction of area above the threshold was measured.

### SiR-DNA staining and live-cell imaging

Forty-eight hours before imaging, cells were seeded in six- or 12-well glass-bottom plates (Cellvis). On the day of imaging, cells were stained with 125 nM SiR-DNA or Spy650-DNA (Spirochrome) in culture medium for 3 h and afterward maintained in Opti-MEM supplemented with 12.5 nM SiR-DNA or Spy650-DNA and the indicated drugs during microscopy. For a few cell lines (explicitly labeled in the figure legends), 10 µM verapamil (Spirochrome, from SiR-DNA kit) was added to the staining and imaging medium to increase SiR-DNA incorporation. Automated microscopy of dividing human cells was performed on a Celldiscoverer 7 screening microscope (Zeiss) using a 50× water immersion objective (Zeiss plan-apochromat 50×/1.2-W autocorr and autoimmersion objective) with a 0.5× zoom lens and recorded as a three-dimensional time series. Each time series was 2.5 h long, with images taken every 3 min. Imaging was controlled by Zen 2.5 (blue edition) software. Cells were maintained at 37°C in a humidified atmosphere of 5% CO_2_ throughout the entire experiment.

### RNAi

The siRNA (Dharmacon) used in this study is listed in Supplemental Table S7. A nontargeting scrambled siRNA was used as a negative control. siRNA transfections were performed using Lipofectamine RNAiMAX (Thermo Fisher) according to the manufacturer's instructions and at room temperature. Diploid HAP1 and RPE1 cells were plated at 25%–30% confluence 12 h prior to siRNA transfection in six-well glass-bottom plates. Nine microliters of RNAiMAX was diluted in 150 µL of Opti-MEM (Gibco), and 3 µL of 10 µM siRNA (30 pmol) was dissolved in 150 µL of Opti-MEM. Both solutions were combined, mixed by pipetting, and incubated for 5 min. The siRNA–lipid complex was then added dropwise to six wells, and cells were incubated for 24 h in the presence of the siRNA. Cells were subsequently subjected to SiR-DNA staining and live-cell imaging as described above. Nocodazole was used at 250 ng/mL as a readout for siRNA efficacy.

### DNA content analysis by flow cytometry

Human cells were trypsinized and stained with 0.2 µg mL^−1^ Hoechst 33342 (Thermo Fisher Scientific) for 30 min at 37°C. Analysis by flow cytometry immediately after incubation was done on a BD FACSAria IIu cell sorter (BD) using an argon laser tuned for UV (353–365 nm) and fluorescence detection at 480 nm.

### Fluorescence-activated cell sorting (FACS)

FACS was performed on a BD FACSAria IIu cell sorter (BD) or a BD FACSMelody cell sorter (BD). For all cell lines and FACS experiments, medium containing PBS and 10% FBS was used. FACS was performed either in bulk or single-cell-sorted into 96-well plates depending on the experiment. Growth medium for FACS-sorted cells contained normocin (Fisher Scientific) following the first 2 d after sorting to suppress mycoplasma, bacterial, and fungal contamination.

### p53 function assay and immunostaining

*p53*^+/+^ and *p53*^−/−^ cell lines were treated with 6.22 Gy γ-irradiation (Co60-Dmax), collected, and fixed in ice-cold ethanol for at least 20 min. After ethanol removal, fixed cells were washed twice with PBS and then permeabilized with 0.25% Triton X-100 in PBS for 10 min at 4°C. Cells were blocked for 1 h with 1% BSA in PBS and stained for 2 h with a mouse monoclonal MPM-2 antibody (10 µg/mL; Abcam). After several washes, cells were stained with 10 µg/mL Alexa 488-conjugated goat antimouse secondary antibody (Thermo Fisher Scientific). DNA was stained with propidium iodide-RNase solution (PI; final concentration of 50 µg/mL + 100 µg/mL RNase A in PBS) for another 20 min in the dark at room temperature and then subjected to FACS analysis.

### Engineering of full and partial chromosome arm deletions

For the engineering of chromosome arm deletions, the CRISPR/Cas9 system (Ran et al. 2013) was used based on an approach adapted from Zuo et al. (2017). Guide RNAs that target repetitive sequences within the chromosome arm were selected based on a custom-made Python script. Additionally, single cutting sgRNAs targeting the intergenic region either close to the centromere or at the respective partial deletion sites were selected based on their top ranking in the online tool GuideScan (https://guidescan.com [Perez et al. 2017], homepage recently updated to GuideScan2; Schmidt et al. 2022) and CRISPOR (http://crispor.tefor.net; Concordet and Haeussler 2018). Off-target analysis was performed using Cas-OFFinder (CRISPR RGEN tools, http://www.rgenome.net/cas-offinder; Bae et al. 2014). All sgRNAs used for engineering of whole or partial chromosome deletions are listed in Supplemental Table S3 and Supplemental Figure S10B. The number of protein-coding genes was calculated based on the MANE project (release v1.0; Morales et al. 2022).

For full and partial chromosome arm deletions, dipHAP1 *p53*^−/−^ cells were seeded subconfluently in a six-well plate 1 d before transfection. One microgram of pSpCas9(BB)-2A-GFP plasmid was transfected with XtremeGene9 (Roche). Forty-eight hours after transfection, cells were FACS-sorted for GFP expression either in bulk or as single cells in 96-well plates. Three days later, bulk-sorted cells were single-cell-sorted into 96-well plates. Cell clones were expanded to confluency in a 12-well plate. Genomic DNA was extracted with 0.5× lysis buffer (Ramlee et al. 2015), and DNA concentration was measured with the Qubit dsDNA HS assay (Invitrogen) according to the manufacturer's protocol. The generation of aneuploidy was ascertained by quantitative PCR (qPCR) according to a modified protocol from Ravichandran et al. (2018). In brief, Luna universal qPCR master mix (NEB) and 10 ng of DNA of each sample were used together with two primer pairs targeting the telomeric and centromeric regions of the respective chromosome arms (Supplemental Fig. S10B; Supplemental Table S4). All qPCR reactions were performed on Mastercycler ep Realplex platforms (Eppendorf). Each calculated cycle threshold (*Ct*) value was normalized to the *Ct* value generated by a primer pair targeting the euploid chromosome 4 (ALB control primer). Samples and controls were measured in triplicates. In addition to qPCR analysis, the karyotypes of all engineered aneuploid cell lines were verified by whole-genome sequencing according to the above-described protocol. The aneuploid cell lines were frozen in multiple aliquots, and for each experiment, fresh aliquots were thawed and cultivated for a maximum of six passages to reduce the formation of secondary karyotypic changes.

### Cocultivation assay

GFP-expressing and nonfluorescent (dark) or mCherry-expressing cell lines were seeded separately in 10-cm dishes prior to FACS sorting. The two cell lines were then mixed in a 50:50 ratio by bulk-sorting 400,000 cells each into 4 mL of growth media with 1:500 normocin. Setting up the gates was done using a nonfluorescent (dark) parental cell line and GFP- or mCherry-expressing cell lines as controls (Supplemental Figs. S11B, S13F). Mixed cell lines were cultured in standard growth medium or growth medium supplemented with 300 nM reversine in three or six replicates for each growth condition, and 50,000 mixed cells were added into each well of a six-well plate. The remaining mixed cells were used to determine the exact distribution of GFP^+^ and dark cells (or mCherry^+^) at day 0 by flow cytometry. After 4 d, cell mixtures were passaged, and the relative abundance of GFP was measured by flow cytometry. All later measurements were done in intervals of 3–4 d for a total duration of 13–19 d.

### Western blotting

For protein preparation, 2 million or 4 million cells were lysed in 75 µL of ice-cold NP40 buffer with cOmplete protease inhibitor cocktail (Roche) and incubated for 1 h on ice. Samples were then incubated with 0.1 µL of benzonase nuclease (Sigma) for 30 min at 37°C. Twenty-five microliters of 4× protein sample buffer was added and boiled for 5 min at 95°C. Protein size was estimated using the prestained protein Ladder midrange molecular weight (Abcam). Coomassie staining was used to equalize the amount of each sample for loading. SDS-PAGE was performed at 120 V for 90 min. Wet transfer onto a nitrocellulose membrane was performed at 250 mA for 150 min at 4°C. Membranes were blocked for 1 h in 5% BSA (Sigma) or 5% skim milk (Gerbu Biotechnik GmbH) in Tris-buffered saline with 0.01% Tween (TBST), depending on the primary antibody (see below). Primary antibodies diluted in the respective blocking buffers were incubated overnight at 4°C on an orbital shaker. The membranes were washed for 30 min in TBST with three buffer exchanges before and after secondary antibody incubation. Horseradish peroxidase (HRP)-conjugated antimouse IgG secondary antibody (1:10,000; Cell Signaling Technology 7076) diluted in the respective blocking buffers was incubated for 1 h at room temperature. Chemiluminescence was developed using the Amersham ECL Prime detection reagent (Cytiva) and detected with the ChemiDocMP imaging system (Bio-Rad). Quantification of protein bands was carried out using ImageJ. The following primary antibodies were used: anti-CDC16 (in skim milk–TBST; 1:500; mouse; Santa Cruz Biotechnology [E-4] sc-365636), anti-p31^comet^ (in BSA–TBST; 1:500; mouse; Sigma-Aldrich E29.19.14), and anti-α-Tubulin (in BSA and skim milk–TBST, respectively; 1:20,000; mouse; Sigma-Aldrich T6074).

### CRISPRa overexpression

To generate dCas9-VPR-mCherry-expressing dipHAP1 cell lines for CRISPRa, HEK293HiEx cells were transfected using XtremeGene9 (Roche) with an envelope plasmid, packaging plasmid, and dCas9-VPR-mCherry expression plasmid for lentivirus production (a gift from Anna Obenauf, IMP Vienna). The expression plasmid had both dCas9-VPR and mCherry under the EF1α promoter separated by P2A. After 3 d, the supernatant-containing virus was filtered through a 0.45-µm filter, and the cell lines were transduced with a 1:1 dilution of the virus. After 3 d of incubation, the cells were FACS single-cell-sorted for mCherry fluorescence. Clonal colonies were expanded, and cell lines stably expressing a moderate to high level of mCherry were selected.

For the induction of overexpression, the dCas9-VPR-mCherry cell lines were transfected with GFP-expressing sgRNA plasmids containing guides targeting the promoter of the gene of interest or the empty vector as a control using XtremeGene9 (Roche). The guide sequences are listed in Supplemental Table S8. For the overexpression of p31^comet^, three sgRNAs were used together. Plasmid-expressing cells were selected with 1.5–2 mg/mL G418. After at least 5 d of selection, mitotic timing was measured as described above, and cells were pelleted for RT-qPCR to measure the change in mRNA expression levels.

For RT-qPCR, RNA was isolated using the RNeasy mini kit (Qiagen). Ten micrograms of RNA was treated with RNase-free DNase I (NEB). cDNA was generated following the ProtoScript II reverse transcriptase (NEB) protocol with oligo d(T)23VN (NEB) and RNasin ribonuclease inhibitor (Promega). cDNA concentration was measured with the Qubit dsDNA HS assay (Invitrogen), and samples were normalized to the same concentration for RT-qPCR (4–8 ng). Expression levels were measured with the same qPCR protocol as described above. All qPCR reactions were performed on Mastercycler ep Realplex platforms (Eppendorf). Cycle threshold (*Ct*) values were normalized to the *Ct* value generated by a primer pair targeting *GAPDH*. Samples and controls were measured in triplicate. The RT-qPCR primers used are listed in Supplemental Table S9.

### Statistical analysis and sample number

All experiments were repeated multiple times, and the indicated number of experiments always refers to biological replicates. Statistical analysis was performed using Python 3.4 and GraphPad Prism software. For statistical analysis of differences versus the wild-type cell lines, the unpaired Student's *t*-test was used. Details for the statistical tests used in a particular experiment are reported in the figure legends. Error bars represent ±standard deviation (SD) unless otherwise indicated.

## Supplementary Material

Supplemental Material
