# Supplementary material for: Adaptation to spindle assembly checkpoint inhibition through the selection of specific aneuploidies
Source: Genes Dev. 2023 Mar 1;37(5-6):171–90. doi: 10.1101/gad.350182.122 (PMC10111865; doi:10.1101/gad.350182.122)
Supplement: Supplemental Material [file supp_37_5-6_171__DC1.html]

Adaptation to spindle assembly checkpoint inhibition through the selection of specific aneuploidies — Adaptation to spindle assembly checkpoint inhibition through the selection of specific aneuploidies — Supplemental Material 

# Adaptation to spindle assembly checkpoint inhibition through the selection of specific aneuploidies

## Supplemental Material

- Supplemental\_Fig\_S1.pdf
- Supplemental\_Fig\_S2.pdf
- Supplemental\_Fig\_S3.pdf
- Supplemental\_Fig\_S4.pdf
- Supplemental\_Fig\_S5.pdf
- Supplemental\_Fig\_S6.pdf
- Supplemental\_Fig\_S7.pdf
- Supplemental\_Fig\_S8.pdf
- Supplemental\_Fig\_S9.pdf
- Supplemental\_Fig\_S10.pdf
- Supplemental\_Fig\_S11.pdf
- Supplemental\_Fig\_S12.pdf
- Supplemental\_Fig\_S13.pdf
- Supplemental\_Fig\_S14.pdf
- Supplemental\_Fig\_S15.pdf
- Supplemental\_Tables\_16.01.pdf
