# Supplementary material for: Adaptation to spindle assembly checkpoint inhibition through the selection of specific aneuploidies
Source: Genes Dev. 2023 Mar 1;37(5-6):171–90. doi: 10.1101/gad.350182.122 (PMC10111865; doi:10.1101/gad.350182.122)
Supplement: Supplemental Material [file supp_gad.350182.122_Supplemental_Fig_S1.pdf]

Figure S1

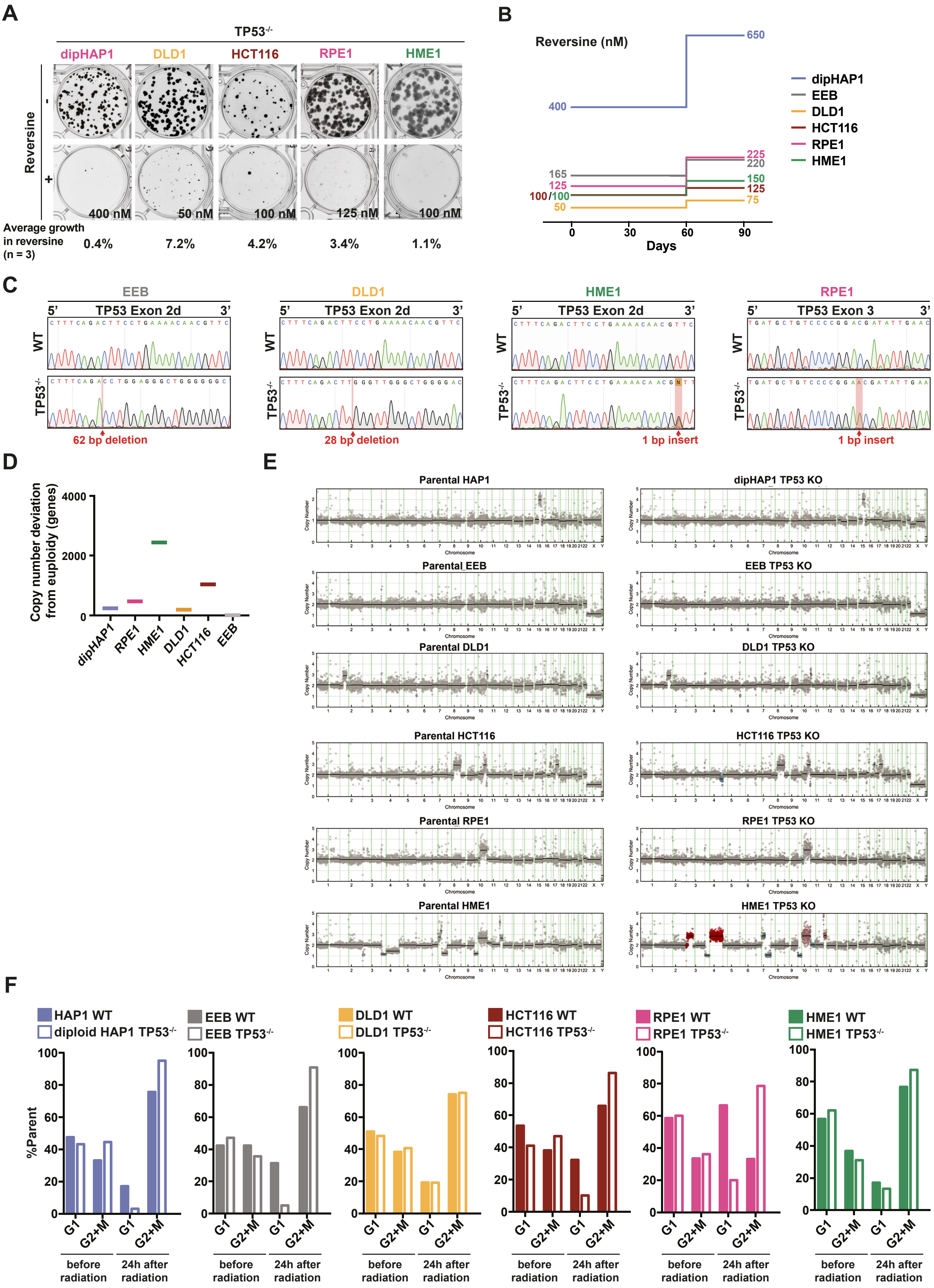
A
TP53-/-
dipHAP1
DLD1
HCT116
RPE1
HME1
Reversine
-
+
400 nM
50 nM
100 nM
125 nM
100 nM
Average growth in reversine (n = 3)
0.4%
7.2%
4.2%
3.4%
1.1%
B
Reversine (nM)
650
400
165
125
100/100
50
225
220
150
125
75
0
30
60
90
Days
dipHAP1
EEB
DLD1
HCT116
RPE1
HME1
C
EEB
DLD1
HME1
RPE1
5' TP53 Exon 2d 3'
5' TP53 Exon 3 3'
WT
TP53-/-
62 bp deletion
28 bp deletion
1 bp insert
1 bp insert
D
Copy number deviation from euploidy (genes)
4000
2000
0
dipHAP1
RPE1
HME1
DLD1
HCT116
EEB
E
Parental HAP1
dipHAP1 TP53 KO
Parental EEB
EEB TP53 KO
Parental DLD1
DLD1 TP53 KO
Parental HCT116
HCT116 TP53 KO
Parental RPE1
RPE1 TP53 KO
Parental HME1
HME1 TP53 KO
Copy Number
Chromosome
F
HAP1 WT
diploid HAP1 TP53-/-
EEB WT
EEB TP53-/-
DLD1 WT
DLD1 TP53-/-
HCT116 WT
HCT116 TP53-/-
RPE1 WT
RPE1 TP53-/-
HME1 WT
HME1 TP53-/-
%Parent
100
80
60
40
20
0
G1
G2+M
before radiation
24h after radiation

Figure S1. A, Growth of *p53* knockout cell lines in the presence or absence of reversine in colony formation assays. Representative images and averaged growth from three independent experiments are shown. **B**, Reversine concentrations that were used during adaptation of the respective cell lines. **C**, DNA sequencing chromatograms of the *TP53* target sites of wildtype and the CRISPR/Cas9 mutated cell lines. **D**, Copy number deviations from euploidy in *p53* knockout cell lines before adaptation as measured by next generation sequencing. Copy number changes are depicted as number of genes. **E**, Copy number profiles of wildtype cell lines and corresponding *p53* knockouts. Shown are copy numbers with each dot representing a 500 kb bin in the genome of cell populations. Segments above 0.5 threshold for gain are colored in red, segments below threshold for loss are colored in blue. Thresholding occurs relative to the parental wildtype. Dotted green lines mark the centromeres in each chromosome. **F**, Bar graphs showing flow cytometric analysis of wildtype (full bar) and corresponding *p53* knockout (empty bar) cell lines before and 24 hours after radiation with 6.22-Gy. Cells were stained with PI and anti-MPM2. Flow cytometry was done as described in the methods. 10,000 cells were analyzed in each experiment. S-Phase percentages are not shown.
