# Supplementary material for: Adaptation to spindle assembly checkpoint inhibition through the selection of specific aneuploidies
Source: Genes Dev. 2023 Mar 1;37(5-6):171–90. doi: 10.1101/gad.350182.122 (PMC10111865; doi:10.1101/gad.350182.122)
Supplement: Supplemental Material [file supp_gad.350182.122_Supplemental_Fig_S2.pdf]

Figure S2

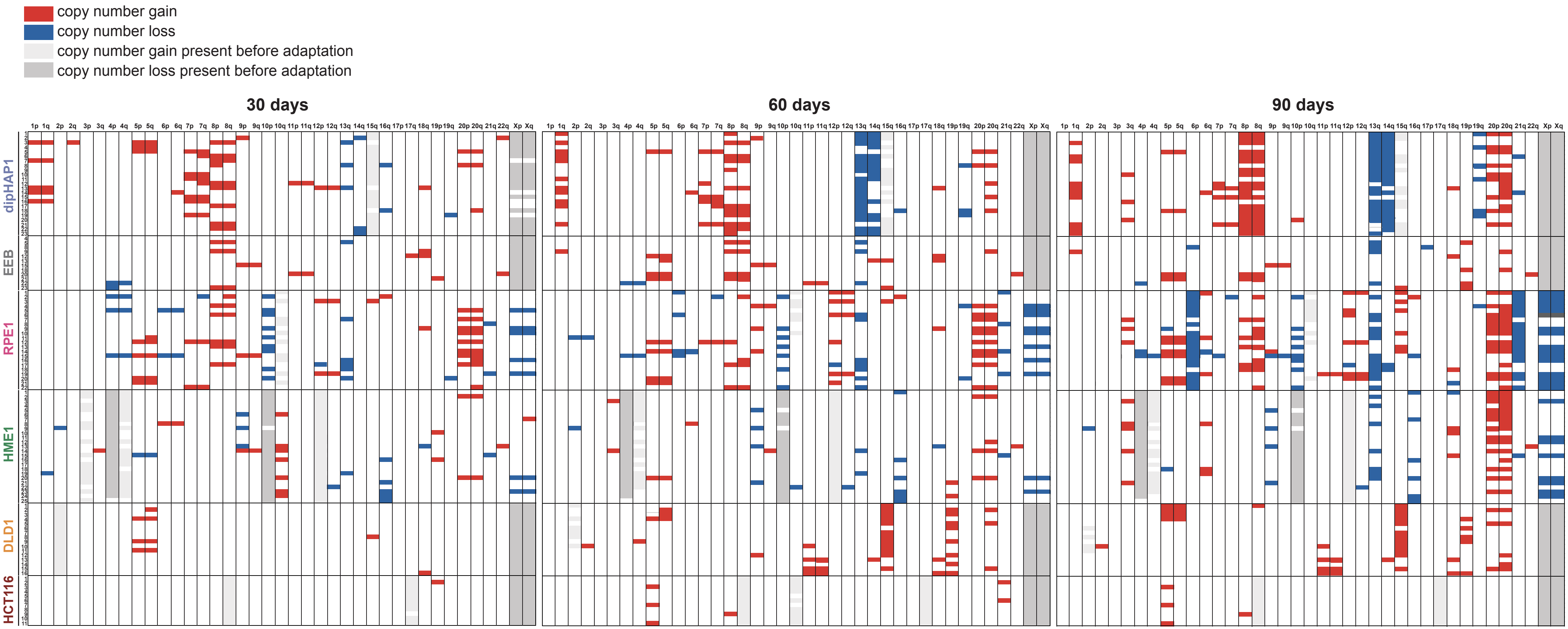

Figure S2. Copy number changes in chromosome arms for the individual adapted populations of each cell line at 30, 60 and 90 days. Cell line specific aneuploidies that were present prior to the adaptation are colored in grey.
