# Supplementary material for: Adaptation to spindle assembly checkpoint inhibition through the selection of specific aneuploidies
Source: Genes Dev. 2023 Mar 1;37(5-6):171–90. doi: 10.1101/gad.350182.122 (PMC10111865; doi:10.1101/gad.350182.122)
Supplement: Supplemental Material [file supp_gad.350182.122_Supplemental_Fig_S3.pdf]

Figure S3

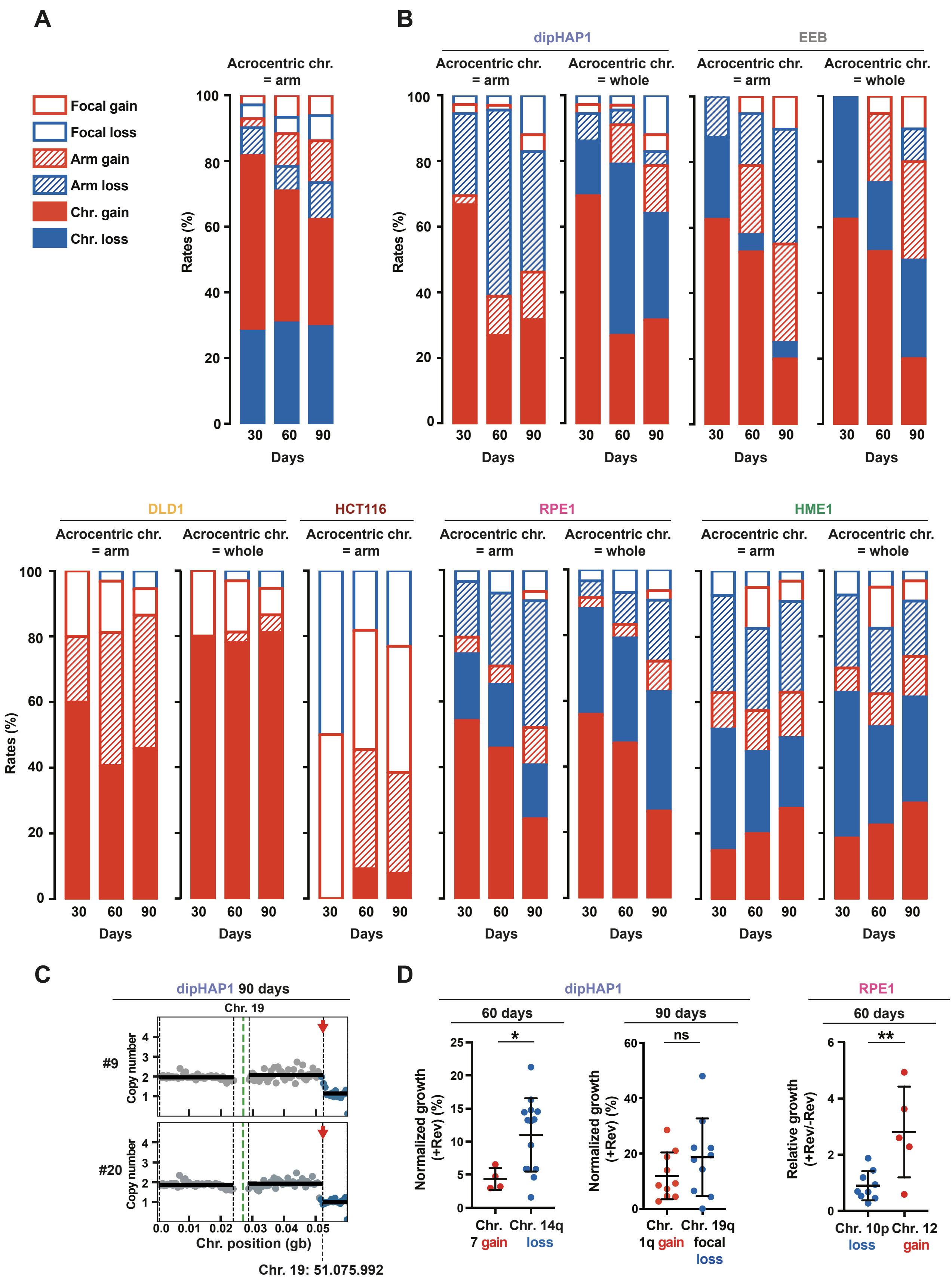

A
Focal gain
Focal loss
Arm gain
Arm loss
Chr. gain
Chr. loss
Acrocentric chr. = arm
Rates (%)
Days
B
dipHAP1
EEB
Acrocentric chr. = arm
Acrocentric chr. = whole
DLD1
HCT116
RPE1
HME1
C
dipHAP1 90 days
Chr. 19
#9
#20
Copy number
Chr. position (gb)
Chr. 19: 51.075.992
D
dipHAP1
RPE1
60 days
90 days
Normalized growth (+Rev) (%)
Relative growth (+Rev/-Rev)
*
ns
**
Chr. 7 gain
Chr. 14q loss
Chr. 1q gain
Chr. 19q focal loss
Chr. 10p loss
Chr. 12 gain

Figure S3. A, Relative proportion of types of copy number changes as in Fig. 1E with acrocentric chromosomes considered as whole-chromosomes instead of arms. **B**, Relative proportion of types of copy number changes of the reversine adapted cell populations at 30, 60 and 90 days from each cell line. For acrocentric chromosomes considered as arm or as whole chromosome. **C**, Copy number profiles of two 90 days adapted dipHAP1 cell lines (#9 and #20) with a 7.5 MB focal loss of chr. 19q. **D**, Comparison of the normalized growth with reversine (+Rev) or relative growth (+Rev/-Rev) of 60 or 90 days adapted dipHAP1 and RPE1 populations with the negatively correlated aneuploidies related to Fig. 1G. p-values are from unpaired student's t-test. * = $p<0.05$, ** = $p<0.01$, ns = not significant.
