# Supplementary material for: Adaptation to spindle assembly checkpoint inhibition through the selection of specific aneuploidies
Source: Genes Dev. 2023 Mar 1;37(5-6):171–90. doi: 10.1101/gad.350182.122 (PMC10111865; doi:10.1101/gad.350182.122)
Supplement: Supplemental Material [file supp_gad.350182.122_Supplemental_Fig_S4.pdf]

Figure S4

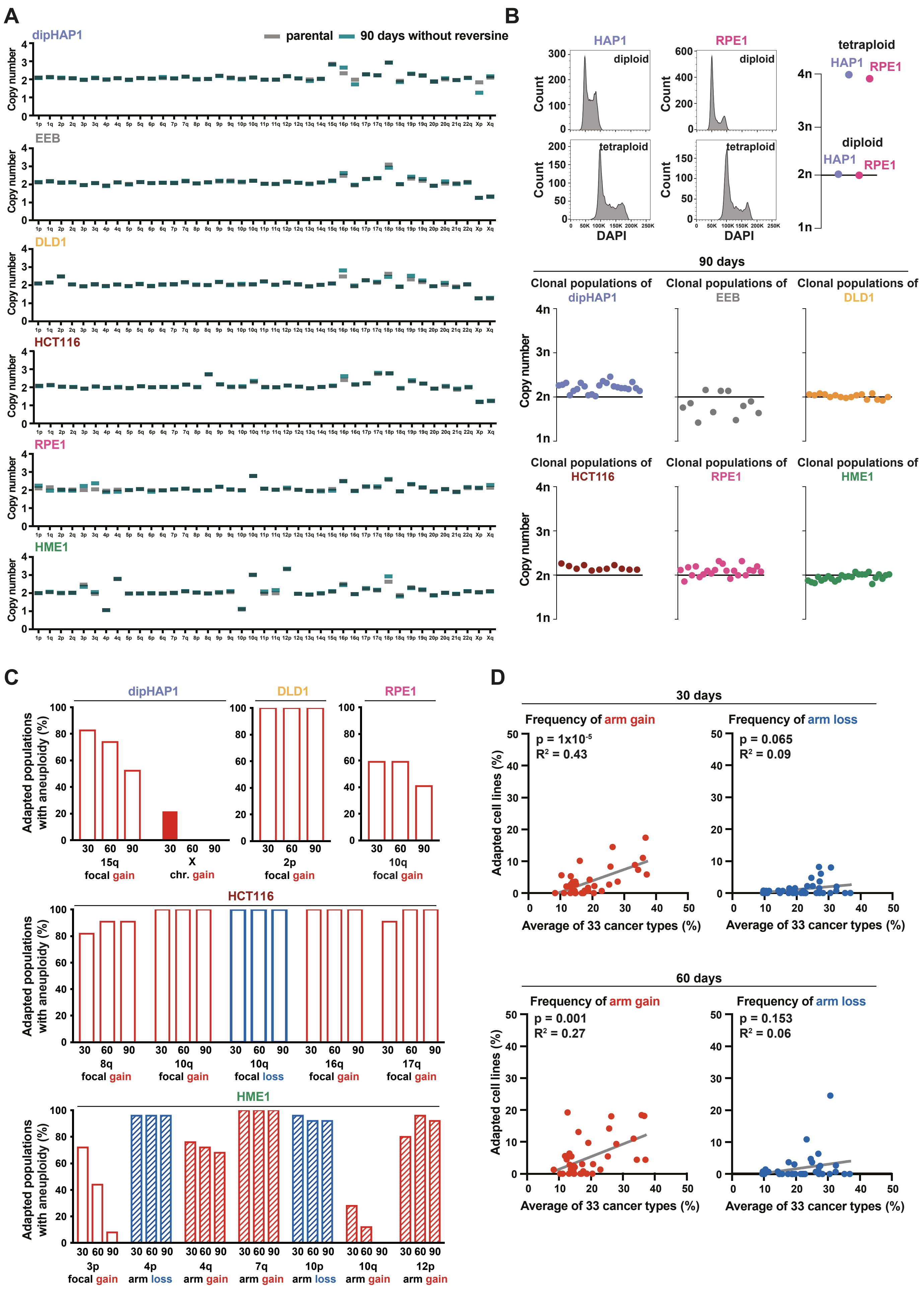

A
dipHAP1
parental
90 days without reversine
Copy number
EEB
DLD1
HCT116
RPE1
HME1
B
HAP1
RPE1
diploid
tetraploid
Count
DAPI
tetraploid
HAP1
RPE1
4n
3n
diploid
HAP1
RPE1
2n
1n
90 days
Clonal populations of dipHAP1
Clonal populations of EEB
Clonal populations of DLD1
Clonal populations of HCT116
Clonal populations of RPE1
Clonal populations of HME1
Copy number
C
dipHAP1
DLD1
RPE1
Adapted populations with aneuploidy (%)
15q focal gain
X chr. gain
2p focal gain
10q focal gain
HCT116
8q focal gain
10q focal gain
10q focal loss
16q focal gain
17q focal gain
HME1
3p focal gain
4p arm loss
4q arm gain
7q arm gain
10p arm loss
10q arm gain
12p arm gain
D
30 days
Frequency of arm gain
p = 1x10-5
R2 = 0.43
Frequency of arm loss
p = 0.065
R2 = 0.09
Adapted cell lines (%)
Average of 33 cancer types (%)
60 days
Frequency of arm gain
p = 0.001
R2 = 0.27
Frequency of arm loss
p = 0.153
R2 = 0.06
Adapted cell lines (%)
Average of 33 cancer types (%)

Figure S4. A, Genome-wide arm copy numbers of parental cell lines before adaptation (time point zero, grey) and after 90 days of cultivation in the absence of reversine (turquoise). 90 days values are an average of six independently cultivated populations. **B**, HOECHST-based flow cytometry analysis of DNA content of diploid and tetraploid dipHAP1 and RPE1 control cell lines (top). G1 DNA content of the reversine adapted populations of all six cell lines at the 90 days time point (bottom). 10,000 cells were analyzed in each measurement; 1n represents haploid DNA content. **C**, Bar graphs depicting the relative abundance of cell line-specific aneuploidies present at time point zero after 30, 60 and 90 days of reversine adaptation. Bar colors depict type of copy number change as in Fig. 1E. **D**, Correlations between the frequencies of chromosome arm gains (red dots) and losses (blue dots) of reversine adapted cell populations at the 30 days and 60 days time points and 33 different cancer types. The R-squared values and p-values from F-tests are depicted in the graphs.
