# Supplementary material for: Adaptation to spindle assembly checkpoint inhibition through the selection of specific aneuploidies
Source: Genes Dev. 2023 Mar 1;37(5-6):171–90. doi: 10.1101/gad.350182.122 (PMC10111865; doi:10.1101/gad.350182.122)
Supplement: Supplemental Material [file supp_gad.350182.122_Supplemental_Fig_S5.pdf]

Figure S5

Cancer type	dipHAP1				EEB				DLD1		HCT116		RPE1				HME1			
	Chromosome gains		Chromosome losses		Chromosome gains		Chromosome losses		Chromosome gains		Chromosome gains		Chromosome gains		Chromosome losses		Chromosome gains		Chromosome losses	
	R squared	P value	R squared	P value	R squared	P value	R squared	P value	R squared	P value	R squared	P value	R squared	P value	R squared	P value	R squared	P value	R squared	P value
ACC	0.0437	0.2013	0.0639	0.1205	0.0886	0.0657	0.1226	0.0289	0.0001	0.9551	0.0276	0.3119	0.1750	0.008	0.0208	0.381	0.0386	0.2308	0.0311	0.2828
BLCA	0.3448	<0.0001	0.0002	0.924	0.0325	0.2719	0.0001	0.9476	0.0011	0.8446	0.0002	0.9334	0.2473	0.0013	0.0006	0.8791	0.2670	0.0008	0.0032	0.7304
BRCA	0.5194	<0.0001	0.0100	0.5456	0.1399	0.019	0.0511	0.1663	0.0001	0.9458	0.0010	0.8484	0.2061	0.0037	0.0040	0.7007	0.1470	0.016	0.1514	0.0144
CESC	0.2404	0.0015	0.0000	0.9791	0.0427	0.2068	0.0583	0.1386	0.0108	0.5283	0.0063	0.6323	0.2048	0.0038	0.0067	0.6206	0.2151	0.0029	0.0010	0.8488
CHOL	0.2268	0.0022	0.0725	0.0973	0.0774	0.0864	0.0732	0.0957	0.0320	0.2759	0.0007	0.8702	0.2066	0.0037	0.0630	0.1233	0.0916	0.0611	0.0640	0.1203
COAD	0.4028	<0.0001	0.0003	0.9122	0.0326	0.2714	0.0008	0.8669	0.0046	0.6833	0.0033	0.7279	0.2861	0.0005	0.0076	0.5966	0.2543	0.0011	0.0376	0.2372
DLBC	0.0112	0.5206	0.0093	0.5593	0.0000	0.992	0.0012	0.8361	0.0071	0.6089	0.0106	0.5324	0.0006	0.8829	0.0195	0.3961	0.0025	0.7647	0.0001	0.965
ESCA	0.5252	<0.0001	0.0060	0.6401	0.0339	0.262	0.0228	0.3592	0.0115	0.5168	0.0033	0.7275	0.3202	0.0002	0.0333	0.2659	0.2191	0.0027	0.0261	0.3262
GBM	0.0566	0.1446	0.0198	0.3933	0.0015	0.8142	0.0082	0.5832	0.0027	0.7517	0.0044	0.6873	0.0064	0.6289	0.0291	0.2994	0.0697	0.1044	0.0405	0.2193
HNSC	0.4417	<0.0001	0.0011	0.8398	0.1361	0.0208	0.0144	0.4675	0.0105	0.5344	0.0292	0.2983	0.3752	<0.0001	0.0215	0.373	0.1436	0.0173	0.0182	0.4128
KICH	0.0483	0.1786	0.0006	0.8871	0.0205	0.3848	0.0417	0.2124	0.0739	0.094	0.0009	0.8525	0.0823	0.0766	0.1281	0.0253	0.0438	0.2008	0.0115	0.5159
KIRC	0.0252	0.334	0.0532	0.1576	0.0476	0.1823	0.0000	0.9885	0.0171	0.428	0.0425	0.208	0.1295	0.0244	0.0091	0.5636	0.0296	0.2951	0.0000	0.9915
KIRP	0.0024	0.7662	0.0737	0.0946	0.0607	0.1306	0.0055	0.6523	0.0179	0.4168	0.0395	0.2253	0.0063	0.6299	0.0528	0.1594	0.0714	0.1002	0.0027	0.7545
LAML	0.3706	<0.0001	0.0154	0.4524	0.2668	0.0008	0.0019	0.7932	0.0064	0.6275	0.0344	0.2585	0.0686	0.1073	0.0187	0.407	0.0183	0.4111	0.0051	0.6645
LGG	0.0688	0.1068	0.1121	0.0373	0.0337	0.263	0.0328	0.2701	0.0055	0.6533	0.0053	0.6611	0.0010	0.8463	0.0098	0.548	0.0027	0.7531	0.0362	0.2458
LIHC	0.4301	<0.0001	0.0192	0.3999	0.2230	0.0024	0.0424	0.2088	0.0018	0.7959	0.0117	0.512	0.1801	0.0071	0.0040	0.7029	0.0243	0.3433	0.0535	0.1568
LUAD	0.2924	0.0004	0.0229	0.3576	0.0280	0.3091	0.0511	0.1664	0.0004	0.9058	0.0232	0.3545	0.1255	0.0269	0.0085	0.5756	0.0187	0.4063	0.0619	0.1266
LUSC	0.1901	0.0055	0.0250	0.3362	0.0439	0.2006	0.0595	0.1346	0.0047	0.6792	0.0416	0.213	0.2127	0.0031	0.0300	0.2919	0.1601	0.0116	0.0580	0.1396
MESO	0.0471	0.1844	0.1036	0.0457	0.0143	0.4677	0.1037	0.0456	0.0000	0.9906	0.0695	0.105	0.0351	0.2537	0.0150	0.4571	0.0006	0.88	0.0445	0.1973
OV	0.3151	0.0002	0.0120	0.5074	0.0284	0.305	0.0534	0.157	0.0054	0.6564	0.0028	0.7497	0.2837	0.0005	0.0043	0.6921	0.2579	0.001	0.0768	0.0875
PAAD	0.3254	0.0001	0.0074	0.6029	0.0416	0.2128	0.0002	0.9315	0.0048	0.6751	0.0011	0.8383	0.1517	0.0143	0.0445	0.1975	0.0623	0.1255	0.0000	0.9888
PCPG	0.0822	0.0767	0.0051	0.6646	0.0566	0.1448	0.0088	0.5692	0.0012	0.837	0.0023	0.7722	0.0177	0.4193	0.0103	0.5387	0.0011	0.841	0.0301	0.2912
PRAD	0.3250	0.0002	0.0105	0.5356	0.1076	0.0415	0.0263	0.3236	0.0117	0.5128	0.0004	0.8993	0.0749	0.0919	0.0011	0.8441	0.0007	0.8734	0.0891	0.0649
READ	0.3529	<0.0001	0.0011	0.8415	0.0289	0.3008	0.0001	0.9499	0.0143	0.4684	0.0003	0.9217	0.2781	0.0006	0.0042	0.6936	0.3088	0.0002	0.0297	0.2938
SARC	0.0679	0.1091	0.0676	0.11	0.1817	0.0068	0.1141	0.0355	0.0491	0.1752	0.0659	0.1147	0.0862	0.0697	0.0439	0.2005	0.0522	0.1617	0.3538	<0.0001
SKCM	0.4060	<0.0001	0.0009	0.8547	0.0536	0.156	0.0000	0.9719	0.0089	0.568	0.0098	0.5486	0.1200	0.0308	0.0180	0.4156	0.0712	0.1005	0.0320	0.2762
STAD	0.6561	<0.0001	0.0004	0.9035	0.0886	0.0657	0.0054	0.6566	0.0155	0.4509	0.0002	0.94	0.4253	<0.0001	0.0040	0.7018	0.2504	0.0012	0.0001	0.9621
TGCT	0.1349	0.0214	0.0163	0.4393	0.0216	0.3723	0.0800	0.081	0.0133	0.4847	0.0112	0.5207	0.0601	0.1325	0.0030	0.7397	0.0086	0.5738	0.0573	0.1424
THCA	0.0189	0.4038	0.0002	0.9408	0.0080	0.5873	0.0013	0.8297	0.0051	0.665	0.0316	0.2789	0.0061	0.6357	0.0001	0.9547	0.0010	0.8479	0.0031	0.7351
THYM	0.2622	0.0009	0.0050	0.6675	0.1248	0.0274	0.0921	0.0604	0.0598	0.1335	0.0184	0.4102	0.0835	0.0744	0.1751	0.008	0.0065	0.6262	0.0767	0.088
UCEC	0.4962	<0.0001	0.0043	0.6913	0.0667	0.1123	0.0307	0.2859	0.0180	0.4155	0.0081	0.5852	0.1371	0.0203	0.0066	0.6242	0.0639	0.1206	0.0948	0.0566
UCS	0.2902	0.0004	0.0179	0.4162	0.0158	0.4455	0.0281	0.3079	0.0167	0.4337	0.0058	0.6448	0.1236	0.0282	0.0015	0.8147	0.2542	0.0011	0.0600	0.1328
UVM	0.3838	<0.0001	0.0150	0.458	0.1739	0.0083	0.0106	0.5321	0.0024	0.7651	0.0005	0.888	0.1267	0.0262	0.0122	0.5038	0.0028	0.7485	0.0001	0.9499

Figure S5. Simple linear regression correlation analysis of chromosome arm gains and losses of each adapted cell line with TCGA cancer types. The respective R-squared and p-values are depicted. Green shaded cells highlight significant correlations.
