# Supplementary material for: Adaptation to spindle assembly checkpoint inhibition through the selection of specific aneuploidies
Source: Genes Dev. 2023 Mar 1;37(5-6):171–90. doi: 10.1101/gad.350182.122 (PMC10111865; doi:10.1101/gad.350182.122)
Supplement: Supplemental Material [file supp_gad.350182.122_Supplemental_Fig_S6.pdf]

Figure S6

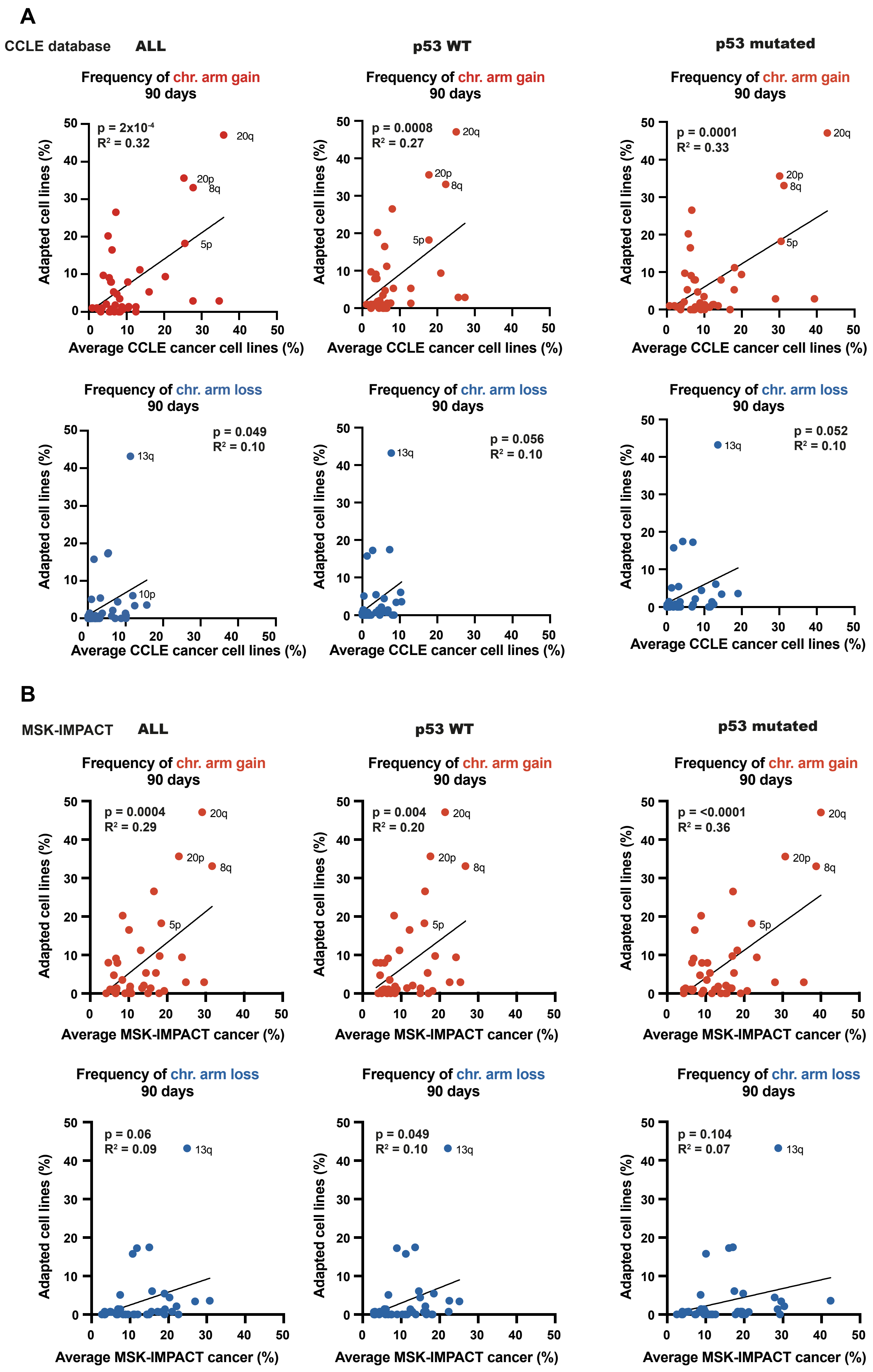
A
CCLE database ALL
p53 WT
p53 mutated
Frequency of chr. arm gain
90 days
p = 2x10-4
R² = 0.32
20q
20p
8q
5p
p = 0.0008
R² = 0.27
p = 0.0001
R² = 0.33
Frequency of chr. arm loss
90 days
p = 0.049
R² = 0.10
13q
10p
p = 0.056
R² = 0.10
p = 0.052
R² = 0.10
Adapted cell lines (%)
Average CCLE cancer cell lines (%)
B
MSK-IMPACT ALL
p = 0.0004
R² = 0.29
p = 0.004
R² = 0.20
p = <0.0001
R² = 0.36
p = 0.06
R² = 0.09
p = 0.049
R² = 0.10
p = 0.104
R² = 0.07
Average MSK-IMPACT cancer (%)

Figure S6. A, Correlation between the frequencies of chromosome arm gains (red dots) and losses (blue dots) in reversine adapted cell populations after 90 days of adaptation and the cancer cell line encyclopedia (CCLE) and **B**, the MSK-IMPACT cancer database. Correlations are depicted for all cancers and cell lines (left row) as well as for cancers and cell lines categorized with wild type p53 (middle row, p53 WT) or with mutated p53 (right row, p53 mutated). Statistical analyses are from simple linear regression.
