# Supplementary material for: Adaptation to spindle assembly checkpoint inhibition through the selection of specific aneuploidies
Source: Genes Dev. 2023 Mar 1;37(5-6):171–90. doi: 10.1101/gad.350182.122 (PMC10111865; doi:10.1101/gad.350182.122)
Supplement: Supplemental Material [file supp_gad.350182.122_Supplemental_Fig_S7.pdf]

Figure S7

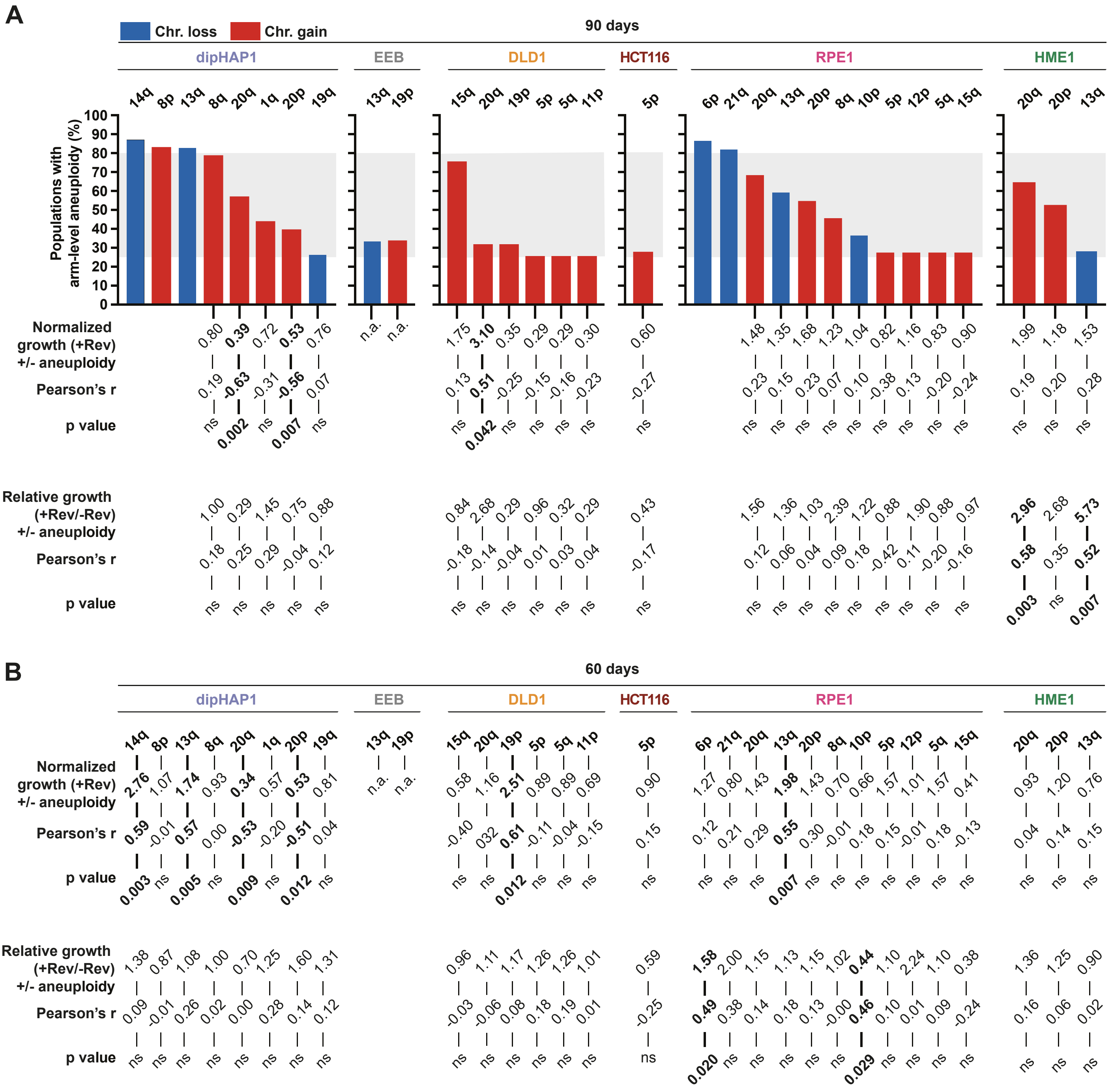

Figure S7. A, Bar graphs representing all arm aneuploidies that are present in >25% of reversine-adapted populations of a given cell type. The grey areas depicts the range (25-80%) in which correlation analysis between copy number changes and fitness was performed for the 90 days time point. Normalized growth with reversine (+Rev) and relative growth (+Rev/-Rev) of the adapted populations with and without the respective aneuploidy as well as corresponding correlation coefficients and p-values are depicted. Growth values were obtained from two independent experiments. **B**, Correlation analysis as in **A**, only for the 60 days time point. p-values are from unpaired student's t-test. ns = not significant. n.a. = not assessed.
