# Supplementary material for: Adaptation to spindle assembly checkpoint inhibition through the selection of specific aneuploidies
Source: Genes Dev. 2023 Mar 1;37(5-6):171–90. doi: 10.1101/gad.350182.122 (PMC10111865; doi:10.1101/gad.350182.122)
Supplement: Supplemental Material [file supp_gad.350182.122_Supplemental_Fig_S8.pdf]

Figure S8

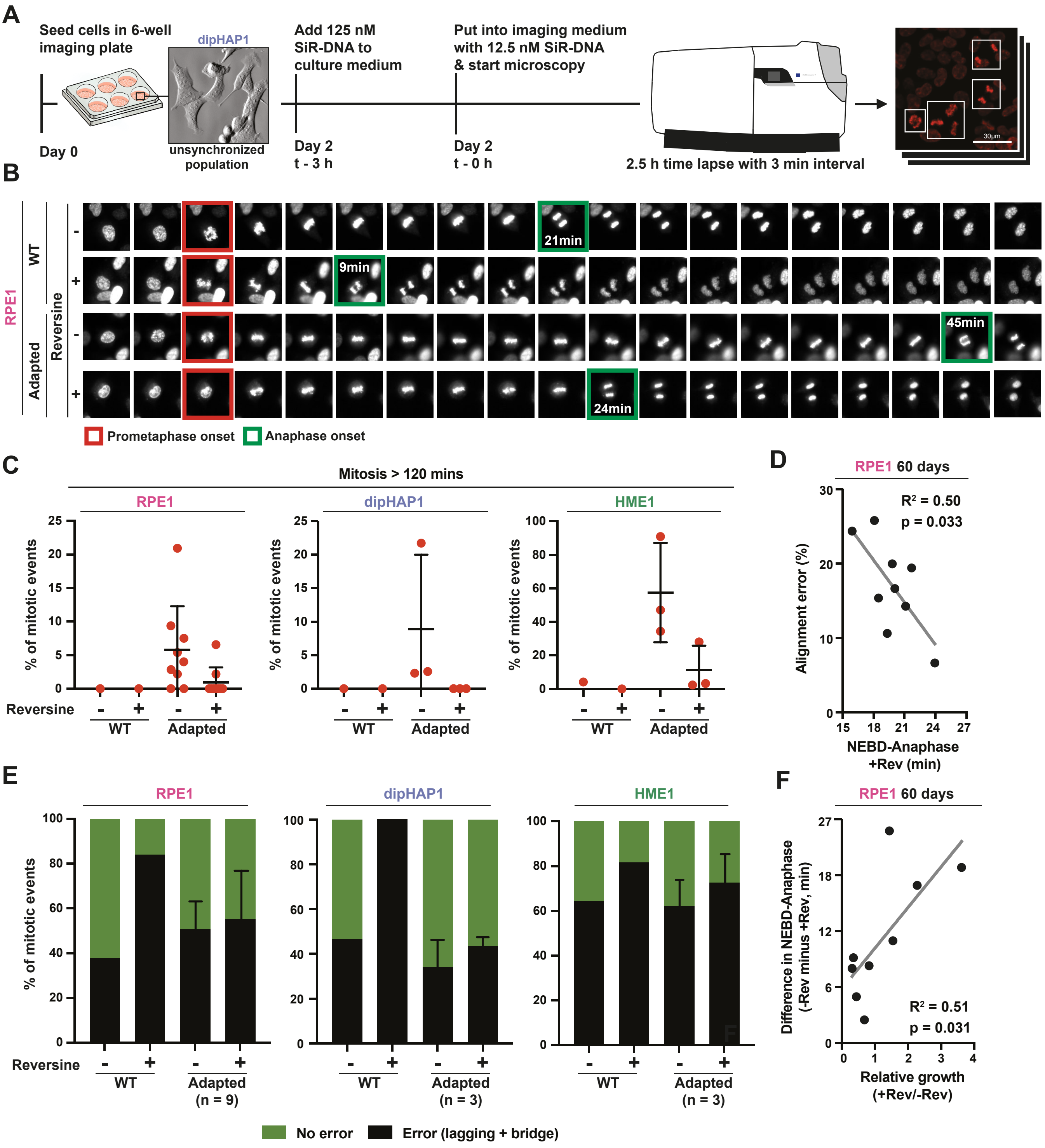
A
Seed cells in 6-well imaging plate
dipHAP1
Day 0
unsynchronized population
Add 125 nM SiR-DNA to culture medium
Day 2
t - 3 h
Put into imaging medium with 12.5 nM SiR-DNA & start microscopy
Day 2
t - 0 h
2.5 h time lapse with 3 min interval
30μm
B
RPE1
WT
Adapted
Reversine
-
+
21min
9min
45min
24min
Prometaphase onset
Anaphase onset
C
Mitosis > 120 mins
RPE1
dipHAP1
HME1
% of mitotic events
Reversine
WT
Adapted
D
RPE1 60 days
R² = 0.50
p = 0.033
Alignment error (%)
NEBD-Anaphase +Rev (min)
E
RPE1
dipHAP1
HME1
% of mitotic events
Reversine
WT
Adapted (n = 9)
Adapted (n = 3)
No error
Error (lagging + bridge)
F
RPE1 60 days
R² = 0.51
p = 0.031
Difference in NEBD-Anaphase (-Rev minus +Rev, min)
Relative growth (+Rev/-Rev)

Figure S8. A, Scheme of the SiR-DNA staining procedure followed by live cell time-lapse imaging. **B**, Full mitotic time-lapse with and without reversine of the parental and adapted RPE1 cells shown in Fig. 4A. **C**, Percentage of mitotic events that lasted longer than 120 min, related to Fig. 4A,B. Each dot represents the mean percentage of an individual adapted population of the respective cell line or the respective parental cell line (WT). **D**, Correlation between the percentage of mitotic events with alignment defects directly preceding anaphase (y-axis) and the NEBD to anaphase duration in reversine (x-axis) of nine 60 days adapted RPE1 population, related to Fig. 4A,C. **E**, Quantification of anaphase errors (bridges and lagging chromosomes) with and without reversine for the unadapted parental cell lines and the adapted cell populations from RPE1 (time point 60 days), dipHAP1 (time point 90 days) and HME1 (time point 90 days). Error bars depict SD between the adapted populations (n depicted in graph) of the respective cell line. **F**, Correlation between the difference in NEBD to anaphase timing with and without reversine (y-axis) and the relative growth (+Rev/-Rev) of the nine 60 days adapted RPE1 populations (x-axis), related to Fig 4A. Analyzed adapted populations are the same as in Fig. 4A-D. p-values are from F-tests.
