# Supplementary material for: Adaptation to spindle assembly checkpoint inhibition through the selection of specific aneuploidies
Source: Genes Dev. 2023 Mar 1;37(5-6):171–90. doi: 10.1101/gad.350182.122 (PMC10111865; doi:10.1101/gad.350182.122)
Supplement: Supplemental Material [file supp_gad.350182.122_Supplemental_Fig_S9.pdf]

Figure S9

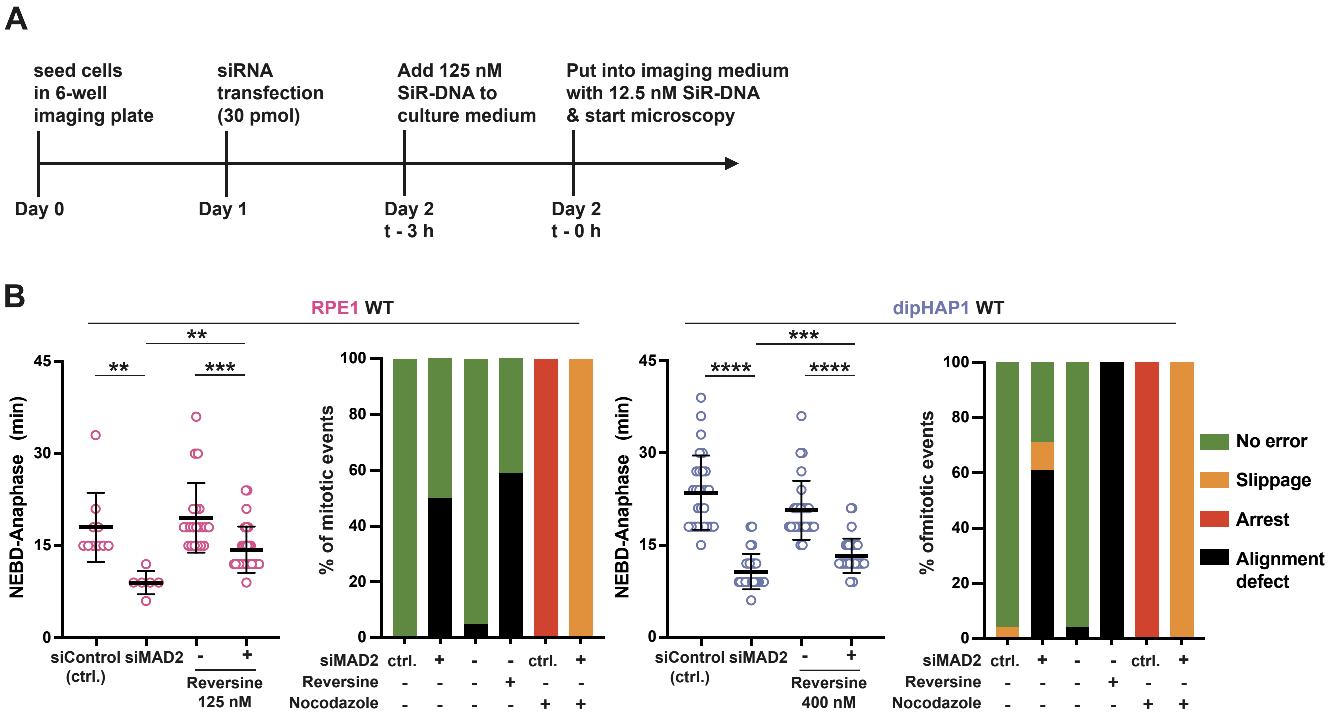

Figure S9. A, Experimental design of the siRNA experiment targeting MAD2 combined with the SiR-DNA live cell imaging set-up. **B**, Quantification of NEBD to anaphase durations and mitotic errors of RPE1 and dipHAP1 unadapted parental (WT) cell lines. Left: Time from NEBD to anaphase in min of WT cell lines transfected either with the indicated siRNA or with and without reversine. Right: Quantification of mitotic errors under the indicated conditions. Green = no error, orange = slippage, red = mitotic arrest, black = alignment defect. p-values are from unpaired student's t-tests, ** = p<0.01, *** = p<0.001, **** = p<0.0001.
