# Supplementary material for: Adaptation to spindle assembly checkpoint inhibition through the selection of specific aneuploidies
Source: Genes Dev. 2023 Mar 1;37(5-6):171–90. doi: 10.1101/gad.350182.122 (PMC10111865; doi:10.1101/gad.350182.122)
Supplement: Supplemental Material [file supp_gad.350182.122_Supplemental_Fig_S10.pdf]

Figure S10

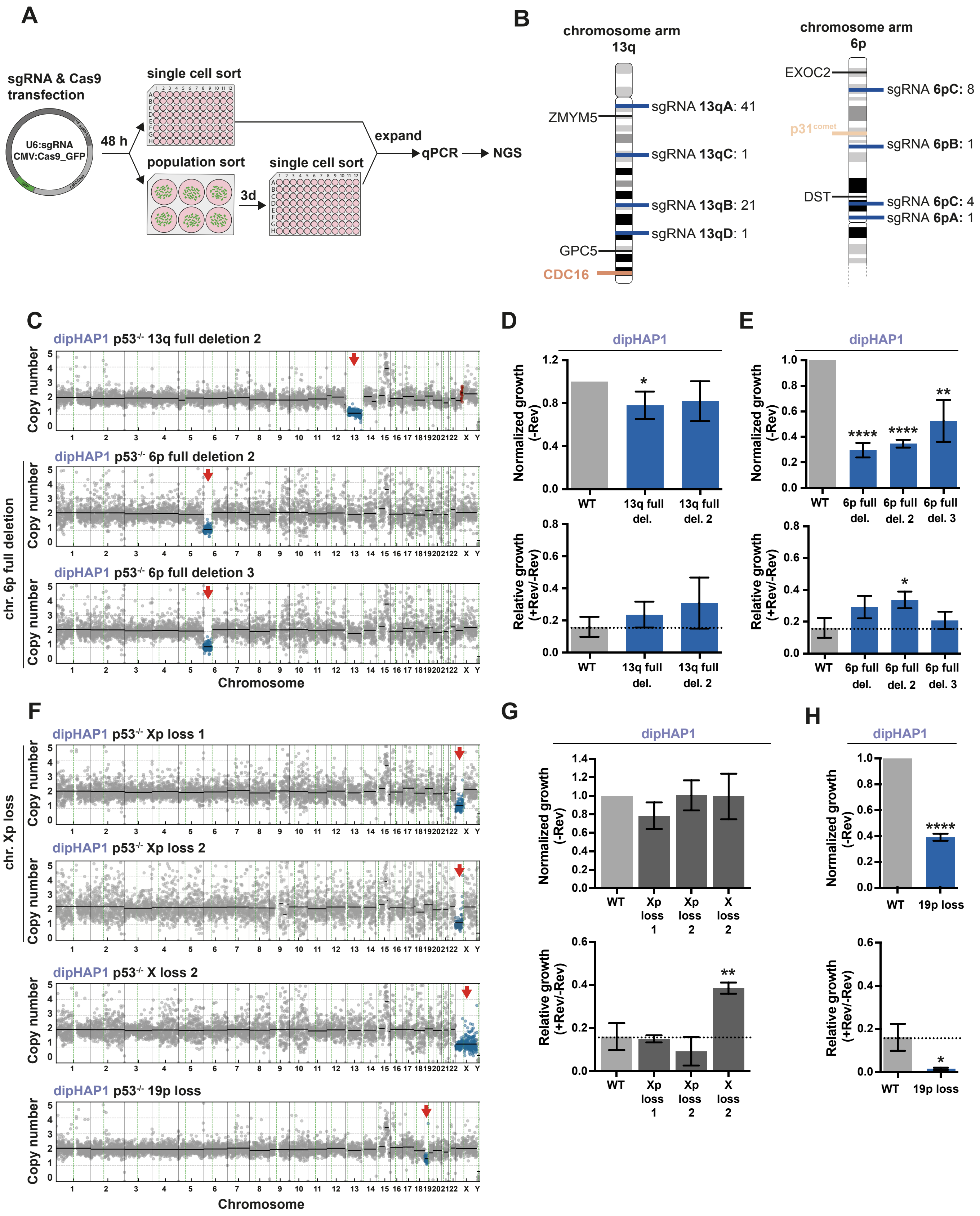

A
sgRNA & Cas9 transfection
U6:sgRNA
CMV:Cas9_GFP
48 h
single cell sort
population sort
3d
single cell sort
expand
qPCR
NGS
B
chromosome arm 13q
ZMYM5
sgRNA 13qA: 41
sgRNA 13qC: 1
sgRNA 13qB: 21
sgRNA 13qD: 1
GPC5
CDC16
chromosome arm 6p
EXOC2
sgRNA 6pC: 8
p31comet
sgRNA 6pB: 1
DST
sgRNA 6pC: 4
sgRNA 6pA: 1
C
dipHAP1 p53-/- 13q full deletion 2
dipHAP1 p53-/- 6p full deletion 2
dipHAP1 p53-/- 6p full deletion 3
chr. 6p full deletion
Copy number
Chromosome
D
dipHAP1
Normalized growth (-Rev)
Relative growth (+Rev/-Rev)
WT
13q full del.
13q full del. 2
E
dipHAP1
6p full del.
6p full del. 2
6p full del. 3
F
dipHAP1 p53-/- Xp loss 1
dipHAP1 p53-/- Xp loss 2
chr. Xp loss
dipHAP1 p53-/- X loss 2
dipHAP1 p53-/- 19p loss
G
dipHAP1
Xp loss 1
Xp loss 2
X loss 2
H
dipHAP1
19p loss

Figure S10. A, Illustration of the experimental procedure to engineer whole and partial chromosome deletions. **B**, Schematic depiction of sgRNA target sites on chromosome arm 13q and 6p (blue lines). Numbers after the semicolon denote the number of target sites on the chromosome arm. The qPCR primer binding locations that were used to screen for successful deletion cell lines are depicted with black lines and the gene name. **C**, Copy number profiles of additional engineered chromosome 13q (13q full del. 2) and 6p deletions (6p full del. 2, 3) in dipHAP1 cells. **D**,**E** Normalized and relative growth of the additional 13q and 6p deletion cell lines measured by colony formation assays. Bars depict mean +/- SD. Dotted line represents mean growth of dipHAP1 WT cell line in reversine. n = 3. **F,** Copy number profiles of the dipHAP1 Xp (Xp loss 1 and 2), additional X loss (X loss 2) and 19p loss cell lines. **G,H** Normalized and relative growth of the dipHAP1 Xp, X loss and 19p loss cell lines measured by colony formation assays. n = 3. p-values are from unpaired student's t-tests, * = $p<0.05$, ** = $p<0.01$, **** = $p<0.0001$. Where not indicated, the p-value was greater than 0.05.
