# Supplementary material for: Adaptation to spindle assembly checkpoint inhibition through the selection of specific aneuploidies
Source: Genes Dev. 2023 Mar 1;37(5-6):171–90. doi: 10.1101/gad.350182.122 (PMC10111865; doi:10.1101/gad.350182.122)
Supplement: Supplemental Material [file supp_gad.350182.122_Supplemental_Fig_S11.pdf]

Figure S11

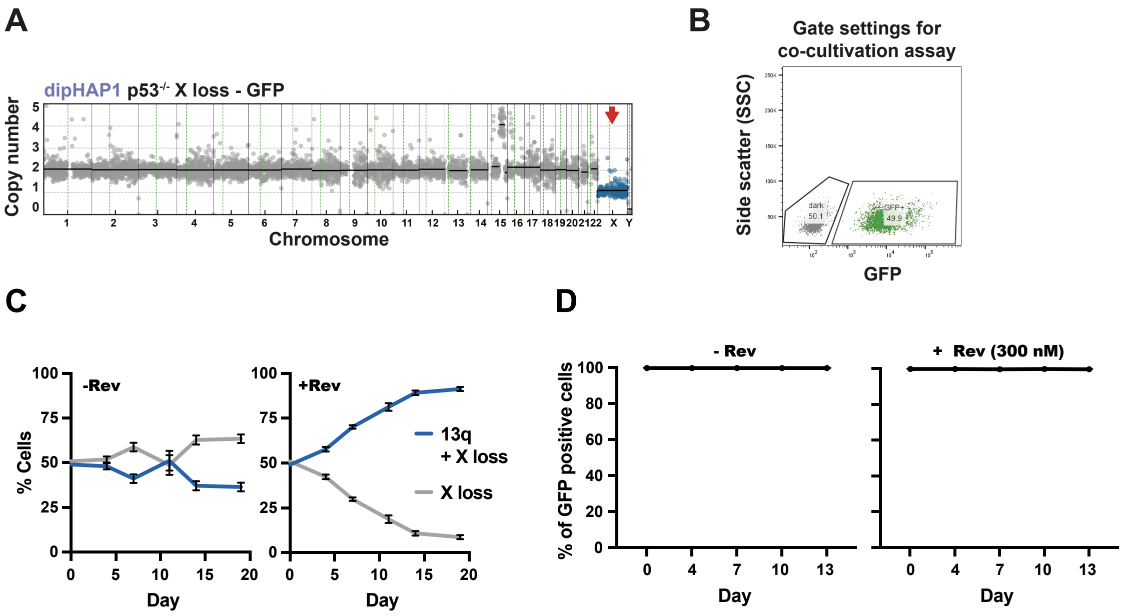

Figure S11. A, Copy number profile of the X loss-GFP cell line that was used for the co-cultivation assay. **B**, Schematic representation of the gates used for the co-cultivation assay both for the initial cell sorting by FACS and the following measurements. **C**, Second independent co-cultivation of the 13q + X loss cell line and the X loss-GFP cell line. The mean +/- SD for 6 separate populations of cells in one time course is shown. **D**, Mean percentage of GFP positive cells of the X loss-GFP cell line without (-Rev) and with (+Rev, 300 nM) over a period of 13 days demonstrating stability of the fluorescent signal over time. The mean +/- SD for 3 separate populations of cells in one time course is shown.
