# Supplementary material for: Adaptation to spindle assembly checkpoint inhibition through the selection of specific aneuploidies
Source: Genes Dev. 2023 Mar 1;37(5-6):171–90. doi: 10.1101/gad.350182.122 (PMC10111865; doi:10.1101/gad.350182.122)
Supplement: Supplemental Material [file supp_gad.350182.122_Supplemental_Fig_S12.pdf]

# Figure S12

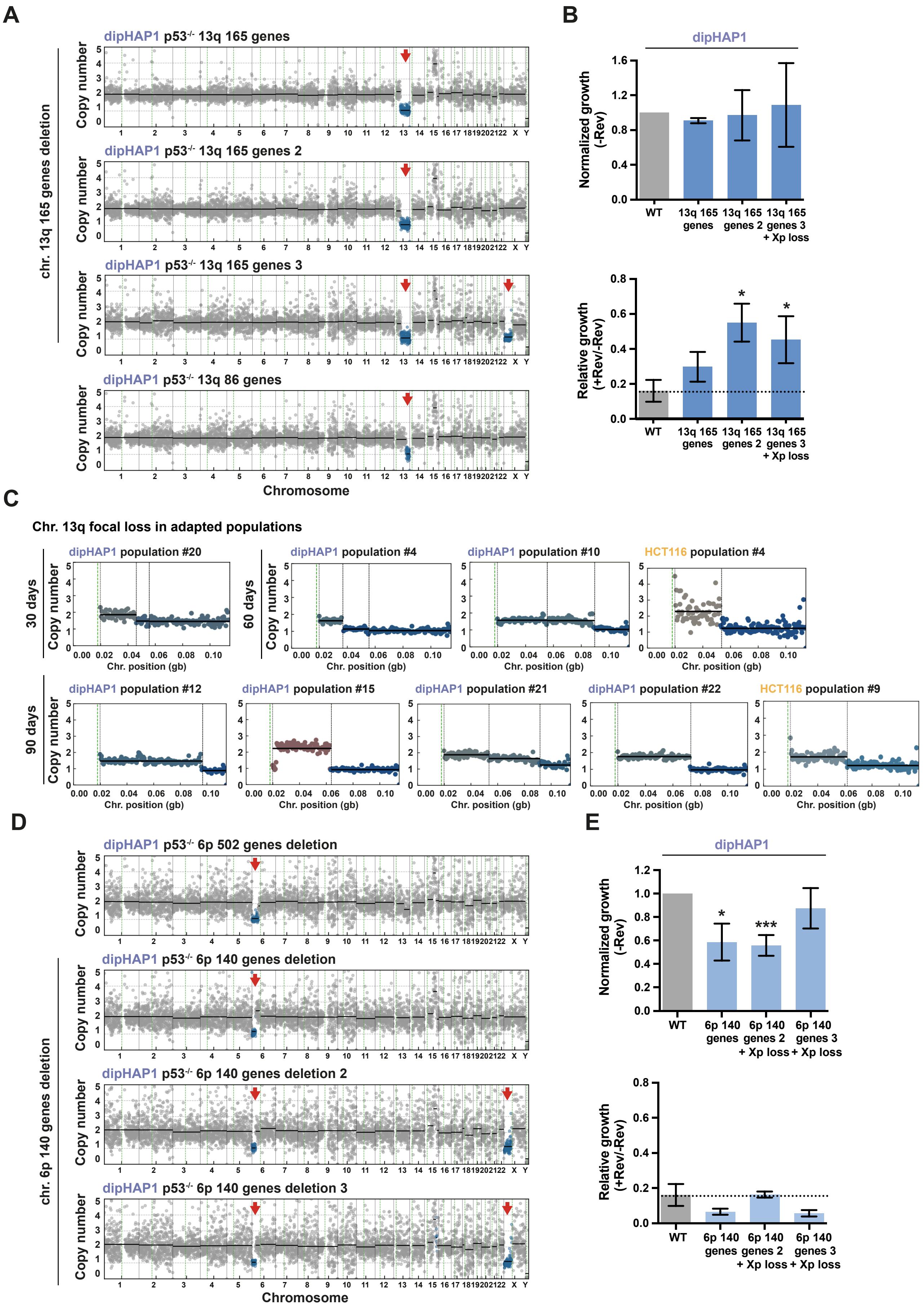
A
chr. 13q 165 genes deletion
dipHAP1 p53-/- 13q 165 genes
dipHAP1 p53-/- 13q 165 genes 2
dipHAP1 p53-/- 13q 165 genes 3
dipHAP1 p53-/- 13q 86 genes
Copy number
Chromosome
B
dipHAP1
Normalized growth (-Rev)
Relative growth (+Rev/-Rev)
WT
13q 165 genes
13q 165 genes 2
13q 165 genes 3 + Xp loss
C
Chr. 13q focal loss in adapted populations
30 days
60 days
90 days
dipHAP1 population #20
dipHAP1 population #4
dipHAP1 population #10
HCT116 population #4
dipHAP1 population #12
dipHAP1 population #15
dipHAP1 population #21
dipHAP1 population #22
HCT116 population #9
Chr. position (gb)
D
chr. 6p 140 genes deletion
dipHAP1 p53-/- 6p 502 genes deletion
dipHAP1 p53-/- 6p 140 genes deletion
dipHAP1 p53-/- 6p 140 genes deletion 2
dipHAP1 p53-/- 6p 140 genes deletion 3
Copy number
Chromosome
E
dipHAP1
Normalized growth (-Rev)
Relative growth (+Rev/-Rev)
WT
6p 140 genes
6p 140 genes 2 + Xp loss
6p 140 genes 3 + Xp loss

**Figure S12. A**, Copy number profiles of all engineered dipHAP1 chromosome 13q partial deletions. Cell line 13q 165 genes 3 also lost chromosome arm Xp. **B**, Normalized and relative growth of the additional 13q partial deletion cell lines (13q 165 genes 2 and 3). Bars depict mean +/- SD. Dotted line represents mean growth of dipHAP1 WT cell line in reversine. n = 3. **C**, Copy number profiles of 13q focal deletions that arose in adapted populations of the dipHAP1 and HCT116 cell lines after 30, 60 or 90 days of adaptation as indicated. **D**, Copy number profiles of all engineered dipHAP1 chromosome 6p partial deletions. Cell lines 6p 140 genes 2 and 3 also lost chromosome arm Xp. **E**, Normalized and relative growth of all 6p partial deletion cell lines. n = 3. p-values are from unpaired student's t-tests, * = $p<0.05$, *** = $p<0.001$. Where not indicated, the p-value was greater than 0.05.
