# Supplementary material for: Adaptation to spindle assembly checkpoint inhibition through the selection of specific aneuploidies
Source: Genes Dev. 2023 Mar 1;37(5-6):171–90. doi: 10.1101/gad.350182.122 (PMC10111865; doi:10.1101/gad.350182.122)
Supplement: Supplemental Material [file supp_gad.350182.122_Supplemental_Fig_S13.pdf]

Figure S13

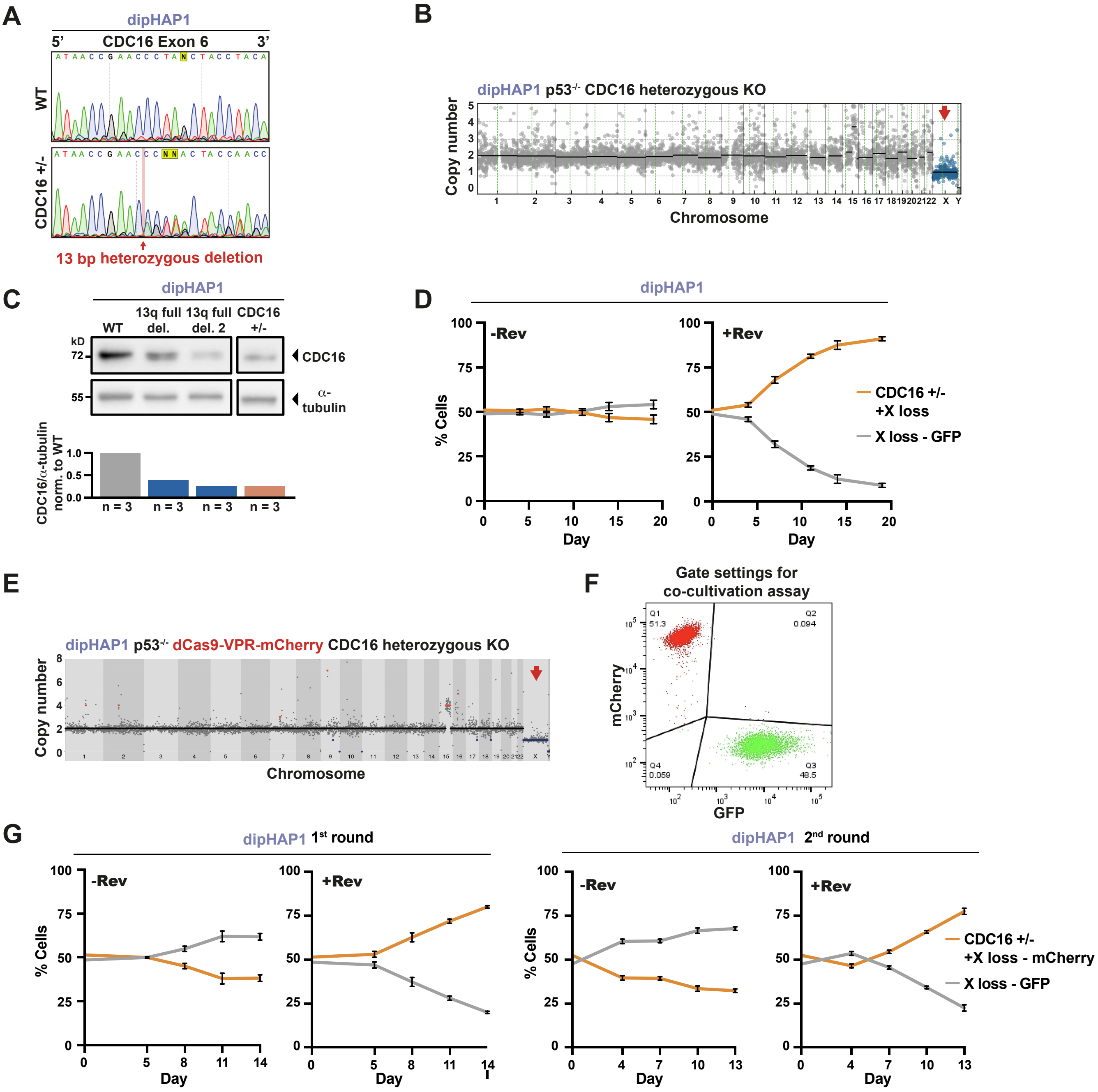

Figure S13. A, DNA sequencing chromatogram of the heterozygous *CDC16* knockout in dipHAP1. **B**, Copy number profile of the dipHAP1 *CDC16*+/- knockout cell line with the additional monosomy of chromosome X. **C**, Western blot of CDC16 protein for the engineered chromosome 13q full deletion and *CDC16*+/- knockout cell lines. Alpha-tubulin was used as a loading control. The mean of the CDC16 intensity relative to the alpha-tubulin intensity and normalized to WT is depicted in the bar graph. **D**, Second independent co-cultivation of the dipHAP1 *CDC16*+/- + X loss cell line and the X loss-GFP cell line, related to Fig. 6C. The mean +/- SD for 6 separate populations of cells in one time course is shown. **E**, Copy number profile of the dipHAP1 dCas9-VPR-mCherry *CDC16*+/- + X loss knockout cell line, which was generated via lentiviral introduction of the dCas9-VPR construct into the dipHAP1 *CDC16*+/- knockout cell line from **B**. **F**, Schematic representation of the gates used for the measurement of the co-cultivation assay of GFP and mCherry labelled cells. **G**, Two independent co-cultivations of the dipHAP1 dCas9-VPR-mCherry *CDC16*+/- + X loss cell line and the X loss-GFP cell line without (-Rev) and with reversine (+Rev). The mean +/- SD for 3 separate populations of cells in one time course is shown.
