# Supplementary material for: Adaptation to spindle assembly checkpoint inhibition through the selection of specific aneuploidies
Source: Genes Dev. 2023 Mar 1;37(5-6):171–90. doi: 10.1101/gad.350182.122 (PMC10111865; doi:10.1101/gad.350182.122)
Supplement: Supplemental Material [file supp_gad.350182.122_Supplemental_Fig_S14.pdf]

Figure S14

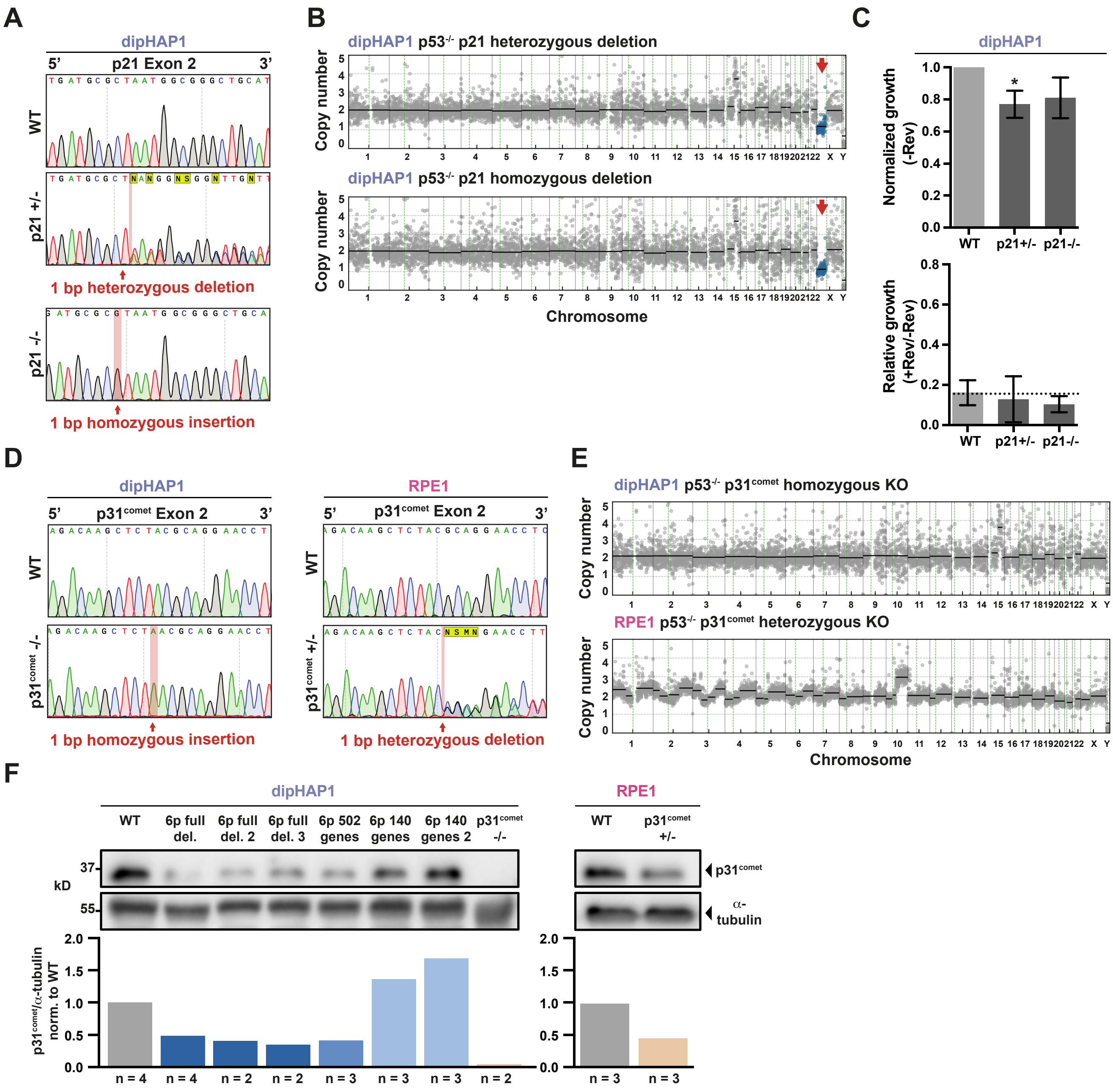

Figure S14. A, DNA sequencing chromatograms of the heterozygous and homozygous *p21* knockout in dipHAP1. **B**, Copy number profiles of the dipHAP1 p*21*+/- and *p21*-/- knockout cell lines with the additional loss of chr. Xp. **C**, Normalized and relative growth of the dipHAP1 *p21*+/- and *p21*-/- knockout cell lines. Bars depict mean +/- SD. Dotted line represents mean growth of dipHAP1 WT cell line with reversine. n = 2. p-values are from unpaired student t-tests, * = p<0.05. Where not indicated, the p-value was greater than 0.05. **D**, DNA sequencing chromatograms of the heterozygous and homozygous *p31^{comet}* knockout in dipHAP1 and RPE1. **E**, Copy number profiles of the heterozygous and homozygous *p31^{comet}* knockout cell lines. **F**, Western blot of p31^{comet} for all engineered chromosome 6p deletion and knockout cell lines. Alpha-tubulin was used as a loading control. Mean value of p31^{comet} intensity relative to alpha-tubulin and normalized to WT is depicted in the bar graph; the respective n values are indicated below.
