# Supplementary material for: Adaptation to spindle assembly checkpoint inhibition through the selection of specific aneuploidies
Source: Genes Dev. 2023 Mar 1;37(5-6):171–90. doi: 10.1101/gad.350182.122 (PMC10111865; doi:10.1101/gad.350182.122)
Supplement: Supplemental Material [file supp_gad.350182.122_Supplemental_Fig_S15.pdf]

Figure S15

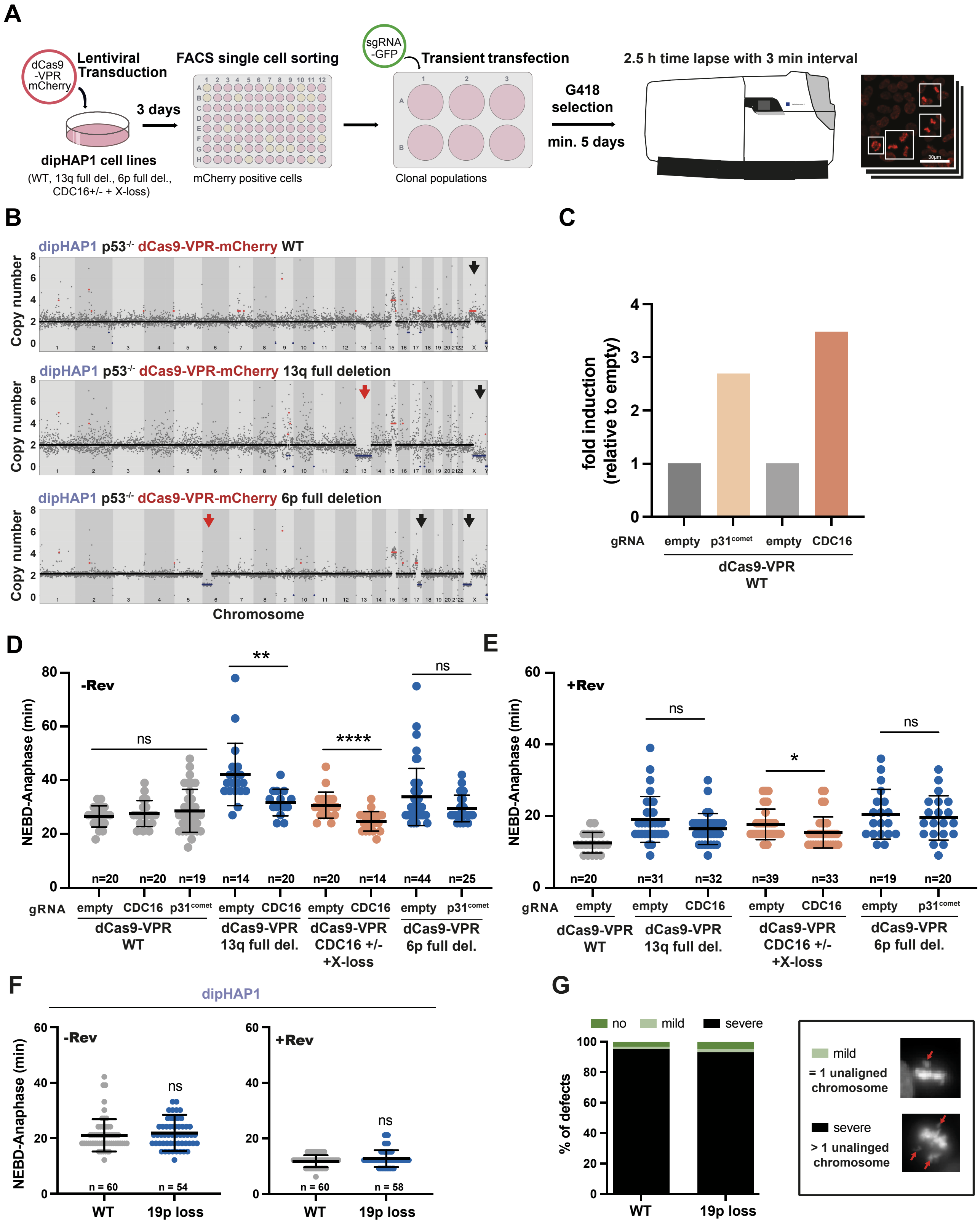
A
dCas9 -VPR mCherry
Lentiviral Transduction
3 days
dipHAP1 cell lines
(WT, 13q full del., 6p full del., CDC16+/- + X-loss)
FACS single cell sorting
mCherry positive cells
sgRNA -GFP
Transient transfection
Clonal populations
G418 selection
min. 5 days
2.5 h time lapse with 3 min interval
B
dipHAP1 p53-/- dCas9-VPR-mCherry WT
dipHAP1 p53-/- dCas9-VPR-mCherry 13q full deletion
dipHAP1 p53-/- dCas9-VPR-mCherry 6p full deletion
Copy number
Chromosome
C
fold induction (relative to empty)
gRNA
empty
p31comet
empty
CDC16
dCas9-VPR WT
D
-Rev
NEBD-Anaphase (min)
ns
**
****
ns
n=20 n=20 n=19 n=14 n=20 n=20 n=14 n=44 n=25
gRNA empty CDC16 p31comet empty CDC16 empty CDC16 empty p31comet
dCas9-VPR WT
dCas9-VPR 13q full del.
dCas9-VPR CDC16 +/- +X-loss
dCas9-VPR 6p full del.
E
+Rev
NEBD-Anaphase (min)
ns
*
ns
n=20 n=31 n=32 n=39 n=33 n=19 n=20
gRNA empty empty CDC16 empty CDC16 empty p31comet
dCas9-VPR WT
dCas9-VPR 13q full del.
dCas9-VPR CDC16 +/- +X-loss
dCas9-VPR 6p full del.
F
dipHAP1
-Rev
+Rev
NEBD-Anaphase (min)
ns
n = 60
n = 54
n = 60
n = 58
WT
19p loss
G
no
mild
severe
% of defects
WT
19p loss
mild = 1 unaligned chromosome
severe > 1 unaligned chromosome

Figure S15. **A**, Schematic overview of the dCas9-VPR CRISPR activation (CRISPRa) of *CDC16* and *$p31^{comet}$*. **B**, Copy number profiles of the lentivirally integrated dCas9-VPR-mCherry cell lines. The 13q full del. and 6p full del. cell lines from Fig. 5A,B were used for integration of the dCas9-VPR-mCherry construct. During the integration process the cell clones acquired secondary copy number changes which are highlighted with black arrows in the profiles, engineered aneuploidies are highlighted with red arrows. **C**, RT-qPCR of the overexpression of *$p31^{comet}$* and *CDC16* in the dCas9-VPR-mCherry WT cell line. n = 1. Fold induction of $p31^{comet}$ and CDC16 is normalized to the empty vector control (empty). GAPDH was used as the reference gene. **D**, Duration from NEBD-anaphase in min for the dipHAP1 dCas9-VPR-mCherry overexpression cell lines without (-Rev), (n = 14-20 mitoses), N = 1, dCas9-VPR 6p full del. + empty: N = 2, and **E**, with reversine (+Rev), (n = 20-39 mitoses), N = 1, dCas9-VPR 13q full del. and CDC16+/- + X-loss: N = 2. The addition of the respective gRNAs for induction of CRISPRa (*CDC16, p31comet*) or the empty vector control (empty) is depicted below the graph. **F**, Duration from NEBD-anaphase in min for the dipHAP1 19p loss cell line without (-Rev), (n = 58-60 mitoses), and with reversine (+Rev), (n = 54-60 mitoses). N = 3. **G**, Quantification of no (dark green), mild (one unaligned chromosome, light green) or severe (multiple unaligned chromosomes, black) alignment defect of the analyzed mitoses from **F**. Example images for the categorization of mitoses into mild (one unaligned chromosome) or severe (multiple unaligned chromosomes) alignment defects. p-values are from unpaired student's t-tests, * = p<0.05, ** = p<0.01, *** = p<0.001, **** = p<0.0001, ns = not significant.
