# Supplementary material for: Adaptation to spindle assembly checkpoint inhibition through the selection of specific aneuploidies
Source: Genes Dev. 2023 Mar 1;37(5-6):171–90. doi: 10.1101/gad.350182.122 (PMC10111865; doi:10.1101/gad.350182.122)
Supplement: Supplemental Material [file supp_gad.350182.122_Supplemental_Tables_16.01.pdf]

Supplemental Tables

Adaptation to spindle assembly checkpoint inhibition through the selection of specific aneuploidies

Manuel Alonso Y Adell[1*], Tamara C. Klockner[1*], Rudolf Höfler[1], Lea Wallner[1], Julia Schmid[1], Ana Markovic[1], Anastasiia Martyniak[1], Christopher S. Campbell[1@]

[1]Department of Chromosome Biology, Max Perutz Labs, University of Vienna, Vienna Biocenter (VBC), A-1030 Vienna, Austria

*: Authors contributed equally
@: Corresponding author
Email: christopher.campbell@univie.ac.at
Phone: +43-1-4277-74418
Fax: +43-1-4277-9562
Address: Dr. Bohr-Gasse 9/5, A-1030 Vienna, Austria

Key words: Aneuploidy Patterns – Chromosomal Instability – Spindle Assembly Checkpoint – Drug Resistance Mechanisms – MPS1

Cell line	Tissue	Phenotype	Sex	Culture Medium	Source
**hTERT-RPE1**	Retina epithelial	Adherent	Female	DMEM (Sigma-Aldrich)<br>+ 2 mmol L-Glutamine (Gibco)<br>+ 1mM Natrium-Pyruvate (Sigma-Aldrich)	I. M. Cheeseman (University of California, USA)
**hTERT-HME1**	Mammary gland epithelial	Adherent	Female	MEBM (Lonza)<br>+ 2 mmol L-Glutamine (Gibco)<br>+ 1mM Natrium-Pyruvate (Sigma-Aldrich)	Evercyte (Cat#: CHT-044-0236)
**DLD1**	Colorectal cancer	Adherent	Male	RPMI-1640 (ATCC) (Sigma-Aldrich)	M. Baccarini (Max Perutz Labs, Austria)
**HCT116**	Colorectal cancer	Adherent	Male	McCoy's 5A (Sigma-Aldrich)<br>+ 2 mmol L-Glutamine (Gibco)<br>+ 1mM Natrium-Pyruvate (Sigma-Aldrich)	B. Vogelstein (The Johns Hopkins Oncology Center, USA)
**diploid HAP1 (dipHAP1)**	Chronic myeloid leukemia (CML)	Adherent	Male	IMDM (Sigma-Aldrich)	J.I. Loizou (CeMM, Austria)
**EEB**	Acute myeloid leukemia (AML)	Suspension	Male	SFEM (STEMCELL Technologies)	Riken (RCB2345)

All culture media were supplemented with 10% FBS and 1% Penicillin-Streptomycin.

Supplemental Table 1. Cell lines used in this study and their culture medium.

Cell line	Karyotype deviations from euploidy
**parental RPE1**	Segmental chr. 10q gain
**RPE1 TP53 KO**	Segmental chr. 10q gain
**parental HME1**	segmental loss of 3p, loss of chr. 4, segmental gain & loss of chr. 7q, chr. 10 isochromosome, chr. 12p gain
**HME1 TP53 KO**	segmental gain of 3p, loss of chr. 4p, gain of chr. 4q, segmental gain & loss of chr. 7q, chr. 10 isochromosome, chr. 12p gain
**parental HAP1**	Segmental chr. 15q gain
**dipHAP1 TP53 KO**	Segmental chr. 15q gain
**parental EEB**	none
**EEB TP53 KO**	none
**parental DLD1**	Segmental chr. 2p gain
**DLD1 TP53 KO**	Segmental chr. 2p gain
**parental HCT116**	Segmental gains of chromosomes 8q, 10q 16q and 17q
**HCT116 TP53 KO**	Segmental gains of chromosomes 8q, 10q, 16q and 17q, segmental 4q loss (above 1.6 threshold)

Supplemental Table 2. Karyotype deviations of the parental cell lines used in this study.

Name	Sequence	Reference
**Chromosome arm 6p deletion**		
**sgRNA 6p A**	ACGGTTTCATTAGTCATACC	This paper
**sgRNA 6p B**	GGGACCGTCACCCTAATAGG	This paper
**sgRNA 6p C**	TGGAATATTGTTCACCTTTA	This paper
**Chromosome arm 13q deletion**		
**sgRNA 13q A**	GGGGGAGTGAATGTGAGTGA	This paper
**sgRNA 13q B**	ATATATGGGGTATACGTATA	This paper
**sgRNA 13q C**	TGGGTTACTTACCGACCGTG	This paper
**sgRNA 13q D**	GATAATACGATAGGCCAGTG	This paper

Supplemental Table 3. sgRNAs that were used for the generation of whole or partial chromosome deletions. Further Illustration in Figure S10 B.

<table>
<tr><th>Name</th><th></th><th>Sequence</th><th colspan="2">Reference</th></tr>
<tr><td colspan="5">Chromosome 6p deletions</td></tr>
<tr><td rowspan="2">EXOC2</td><td>fw</td><td>ATGTCTCGATCACGACAACCC</td><td rowspan="2">PrimerBank, ID: 30581133c1</td><td rowspan="9">Spandidos et al. 2010</td></tr>
<tr><td>rev</td><td>GGCCAGTCCCCAGATTTTCT</td></tr>
<tr><td rowspan="2">DST</td><td>fw</td><td>CTACCAGCACTCGAACCAGTC</td><td rowspan="2">PrimerBank, ID: 291290967c1</td></tr>
<tr><td>rev</td><td>GCCGAAGCTAATGCAAGAGTTG</td></tr>
<tr><td colspan="4">Chromosome 13q deletions</td></tr>
<tr><td rowspan="2">ZMYM5</td><td>fw</td><td>AGAGTTGACTGAACAGACTCCT</td><td rowspan="2">PrimerBank ID: 218083691c1</td></tr>
<tr><td>rev</td><td>GACCAAATGAATCCCCTATGTCC</td></tr>
<tr><td rowspan="2">GPC5</td><td>fw</td><td>GGTGTGACTGACAGTTCCCTG</td><td rowspan="2">PrimerBank, ID: 215272348c3</td></tr>
<tr><td>rev</td><td>TGCAGATAGTCTGTGGTGTTGAT</td></tr>
<tr><td colspan="5">Control primer</td></tr>
<tr><td rowspan="2">ALB
(chr4)</td><td>fw</td><td>TGTTGCATGAGAAAACGCCA</td><td colspan="2" rowspan="2">Bremer et al. 2015</td></tr>
<tr><td>rev</td><td>GTCGCCTGTTCAACCAAGGAT</td></tr>
</table>

Supplemental Table 4. QPCR primers used to identify whole and partial chromosome deletions. Primer binding sites for chr. 6p and 13q are illustrated in Figure S10 B.

Name	Sequence		Reference
p53	fw	CACCGACTTCCTGAAAACAACGTTC	Giacomelli et al. 2018
	rev	AAACGAACGTTGTTTTCAGGAAGTC	
p21	fw	CACCGCCGCGACTGTGATGCGCTAA	McKinley and Cheeseman 2017
	rev	AAACTTAGCGCATCACAGTCGCGGC	
***p31**^{comet}*	fw	CACCGACTTGAGACAAGCTCTACGC	Thu et al. 2018
	rev	AAACGCGTAGAGCTTGTCTCAAGTC	
CDC16	fw	CACCGCTCTAGATAACCGAACCC	This paper
	rev	AAACGGGTTCGGTTATCTAGAGC	

Supplemental Table 5. SgRNAs used in this study for the generation of heterozygous and homozygous knockout cell lines. Underlined sequence represents added overhangs for the creation of dsDNA oligos that can be cloned into the gRNA/Cas9 plasmid, see Methods.

Name	Sequence	
p53	fw	TTATAGGGAGGTCAAATAAGCAGCA
	rev	ATCTACAAGCAGTCACAGCACAT
p21	fw	GCCCGGCCAGGTAACATAGTG
	rev	GTGACAGGTCCACATGGTCTTC
***p31**^{comet}*	fw	GCGTATGTCGGAGTGCCTGC
	rev	GTGCTTAAGCTGTTCATAGG
CDC16	fw	CTATGATCGCACCACTGAACTC
	rev	TGTCAGCATGTGATGTGATGTT

Supplemental Table 6. Genotyping primers used for the identification and analysis of generated knockout cell lines.

Name	Sequence
**Scrambled**	Scramble siRNA (Dharmacon/smartpool format - D-001810-10-05)
**MAD2**	siMAD2 (Dharmacon/smartpool format – L-003271-00-0005)

Supplemental Table 7. SiRNA used in this study for the depletion of MAD2.

Name	gRNA Sequence	Reference
p31^{comet}	GCGTCGCCGCAGTGTGGGGG	This paper, gRNAs were selected using CHOPCHOP, (https://chopchop.cbu.uib.no, (Labun et al. 2019))
	GCGCGGGCCCCGTGCTCAAG	
	GTAACCTAGCGTAGTCACAGT	
CDC16	GTGGGCTTCTGGTTGCCTTG	Horlbeck et al. 2016

Supplemental Table 8. gRNAs used for the overexpression of *p31^{comet}* and *CDC16* via CRISPRa, see Fig. S15A.

Name	Sequence	
p31^{comet}	fw	GAGAAGTCCGAAGAAACTCACG
	rev	CCGAAGCGTTGAGAGGTTCC
CDC16	fw	TCCTGTGTCTTGGTTTGCAG
	rev	CAGAGCTTGGCTGAAGAACC
GAPDH	fw	TTGACCTCAACTACATGGTTTAC
	rev	AGGAGGCATTGCTGATGATC

Supplemental Table 9. RT-qPCR primers used for the identification of the fold change of p31comet and CDC16 in the dCas9-VPR-mCherry WT cell line, see Fig. S15C.

References

Bremer S, Fløisand Y, Brinch L, Gedde-Dahl T, Bergan S. 2015. Glutathione Transferase Gene Variants Influence Busulfan Pharmacokinetics and Outcome After Myeloablative Conditioning: *Therapeutic Drug Monitoring* **37**: 493–500.

Giacomelli AO, Yang X, Lintner RE, McFarland JM, Duby M, Kim J, Howard TP, Takeda DY, Ly SH, Kim E, et al. 2018. Mutational processes shape the landscape of TP53 mutations in human cancer. *Nat Genet* **50**: 1381–1387.

Horlbeck MA, Gilbert LA, Villalta JE, Adamson B, Pak RA, Chen Y, Fields AP, Park CY, Corn JE, Kampmann M, et al. 2016. Compact and highly active next-generation libraries for CRISPR-mediated gene repression and activation. *eLife* **5**: e19760.

Labun K, Montague TG, Krause M, Torres Cleuren YN, Tjeldnes H, Valen E. 2019. CHOPCHOP v3: expanding the CRISPR web toolbox beyond genome editing. *Nucleic Acids Research* **47**: W171–W174.

McKinley KL, Cheeseman IM. 2017. Large-Scale Analysis of CRISPR/Cas9 Cell-Cycle Knockouts Reveals the Diversity of p53-Dependent Responses to Cell-Cycle Defects. *Developmental Cell* **40**: 405-420.e2.

Spandidos A, Wang X, Wang H, Seed B. 2010. PrimerBank: a resource of human and mouse PCR primer pairs for gene expression detection and quantification. *Nucleic Acids Research* **38**: D792–D799.

Thu KL, Silvester J, Elliott MJ, Ba-alawi W, Duncan MH, Elia AC, Mer AS, Smirnov P, Safikhani Z, Haibe-Kains B, et al. 2018. Disruption of the anaphase-promoting complex confers resistance to TTK inhibitors in triple-negative breast cancer. *Proc Natl Acad Sci USA* **115**: E1570-E1577.
